# Interatomic Potentials Transferability for Molecular Simulations: A Comparative Study for Platinum, Gold and Silver

**DOI:** 10.1038/s41598-018-20375-4

**Published:** 2018-02-05

**Authors:** Seyed Moein Rassoulinejad-Mousavi, Yuwen Zhang

**Affiliations:** Department of Mechanical and Aerospace Engineering, University of Missouri, Columbia, Missouri 65211 USA

## Abstract

A perfectly transferable interatomic potential that works for different materials and systems of interest is lacking. This work considers the transferability of several existing interatomic potentials by evaluating their capability at various temperatures, to determine the range of accuracy of these potentials in atomistic simulations. A series of embedded-atom-method (EAM) based interatomic potentials has been examined for three precious and popular transition metals in nanoscale studies: platinum, gold and silver. The potentials have been obtained from various credible and trusted repositories and were evaluated in a wide temperature range to tackle the lack of a transferability comparison between multiple available force fields. The interatomic potentials designed for the single elements, binary, trinary and higher order compounds were tested for each species using molecular dynamics simulation. Validity of results arising from each potential was investigated against experimental values at different temperatures from 100 to 1000 K. The data covers accuracy of all studied potentials for prediction of the single crystals’ elastic stiffness constants as well as the bulk, shear and Young**’**s modulus of the polycrystalline specimens. Results of this paper increase users’ assurance and lead them to the right model by a way to easily look up data.

## Introduction

Atomistic simulations research has been increasingly applied in a wide range of areas, including nanoscience and nanotechnology, especially those of an interdisciplinary nature. The heart of atomistic simulations, such as Molecular Dynamics (MD) or Monte Carlo, is force field or interatomic potential. They define the interaction of atoms in a system and accuracy of results hinge on the choice of these potential. These mathematical functions were fitted per reference data based on materials physical properties for a specific species or compound and in a range of specific properties. These potentials were generated within a certain range of composition, temperature and structure for a specific material and results outside the ranges may be meaningless. The challenge in computational material science at nanoscale is to have a validated model, which has been checked for calculation of an observable property. This would consider the transferability of the potential, a measure of robustness of a model at different conditions other than those used in the fitting process. Because of ambiguities in choice of an appropriate interatomic potential, one often must rely on agreement with experiment as a measure of the accuracy. Effect of temperature range, composition and structure for a specific material as well as the applied fitting model are leading influential factors that affect the transferability and accuracy of a potential. Thus, it is vital to know which interatomic potential works accurately at each of these ranges of fitted properties.

Among multiple existing force fields, EAM potentials represent the most common model of atomic bonding in metallic systems. They have been applied widely to atomistic simulations on nanomaterials properties, particularly in mechanical properties such as elastic deformation, point defects, diffusion, plastic deformation, and fracture. They are popular not only for their mathematic simplicity which makes them conductive to large-scale computer modelling, but also because they are rooted in density-functional theory (DFT). It is important in implementation of interatomic potentials to find their transferability and accuracy in different working temperatures. Therefore, parametrization of different EAM models aimed at developing new force fields with better accuracy has been a recurring theme in the literature. Usually, EAM potentials are constructed by fitting to experimental and/or first-principles data for a single element or compound at zero Kelvin. However, it is not evident that a potential fit to 0 K will be capable to predict a system’s properties at high temperatures. In addition to checking the transferability of potentials, the accuracy of the different created EAM potentials at 0 K must also be considered. Becker *et al*.^1^ considered the effect of multiple EAM interatomic potentials for a single aluminium crystal at 0 K. Kalidindi *et al*.^2^ performed the same studies for the application of data science tools to quantify and distinguish between structures and models in molecular dynamics datasets. Later they provided all the available potentials properties for most of the elements of the periodic table on the National Institute of Standards and Technology (NIST) Interatomic Potentials Repository (IPR) website for zero Kelvin^3^. Now, the challenge is lack of a comparison for performance analysis of these generated potentials as a function of temperature.

The importance of considering different interatomic potentials performance at a finite temperature other than 0 K has been recently studied by Rassoulinejad-Mousavi *et al*.^4^. They investigated several force fields for copper, nickel and aluminium from NIST IPR and LAMMPS databases at room temperature. They examined tens of the potentials at 300 K as a practical temperature for many real-world applications. It was found that some force fields created for an alloy may not be appropriate for all the species existing in the compound. They also concluded that the potentials that were accurate at zero kelvin may not be able to produce the right results at room temperature. Relying on these results, Li and Chew^5^ employed an appropriate force field for nickel to study asymmetrical grain boundary dislocation emission processes observed in MD simulations under applied tensile and compressive loads. They comprehensively discussed the relationship between the traction signatures and periodic structural units along the grain boundary. Similarly, Sun *et al*.^6^ picked their force field for modelling the interaction of the copper atoms in examining the instability of a thin liquid film on nanostructures according to results presented by Rassoulinejad-Mousavi *et al*.^4^ at room temperature. Unfortunately, evaluation of interatomic potentials as a function of temperature has not been considered yet and there is no reference for users to see the capability of each potential at different temperatures.

Due to the lack of a comprehensive work in the literature for considering transferability of interatomic potentials, we were motivated to tackle this problem in the present paper. To do this, elastic properties of cubic single crystals, as prominent features for fitting interatomic potentials, were obtained at different temperatures from 100 to 1000 K. This will result in increasing the atomistic simulations accuracy and introducing robust interatomic potentials with wider applicability for each of the elements Pt, Au and Ag. Because atomistic simulations on these three species are going viral in nanoscale research from biological to engineering applications^7–10^, results of this work pave the way for many investigators worldwide who are applying these species in their molecular simulation research. The data presented here, in easily accessible form, will lead users to employ appropriate models for having high-quality atomistic computer simulations on these three species which are in high demand in nanoscience and nanotechnology studies.

## Simulations Details

MD simulation is used to consider the tensile and shear strains of cubic single crystals of platinum, gold and silver face centred cubic (FCC) lattices, respectively. The clusters were subjected to tensile and shear loads to obtain stress strain behaviour of each nanomaterial using different force fields at various temperatures. The examined temperature range varies from 100 to 1000 K for each potential. Large-scale atomic/molecular massively parallel simulator (LAMMPS)^11^, a classical MD solver, is used for simulations. By use of classical MD at the studied temperatures, one may ask about the importance of quantum zero order effect at low temperatures. The reason for choice of 100 K and above for our MD study is that, this effect previously has been considered by Sheng *et al*.^12^ for our materials under study Pt, Au and Ag. According to comparisons they made, it was shown that discrepancies between results of MD simulation and those evaluated by considering zero-point energy, are negligible above 100 k for all elements studied here. Thus, this should be a safe range of temperature range for present MD simulations, and one can confidently say quantum zero order effects could not become important in this work. Cubic boxes with dimensions of 50*a* (*a* is lattice constant) are created for platinum, gold and silver. The reason for having a large simulation box is to avoid thermal perturbations of a perfect lattice. On the other hand, larger simulation cell sizes should be used to converge the dislocation nucleation stress values and to not influence the dislocation nucleation mechanism. The periodic boundary condition is applied in the x-, y-, and z-directions. Distinctive styles of the EAM potential is adopted in the simulations. The potential energy of an embedded atom *i* can be approximated as follows^13^,

1 $${E}_{tot}=\sum _{i}{F}_{i}({\rho }_{h,i})+\frac{1}{2}\sum _{i}\sum _{j(\ne i)}{\varphi }_{ij}({R}_{ij})$$

where *F*_*i*_(*ρ*_*h,i*_) is the embedding energy for embedding atom *i* into the host electron density *ρ*, and *ϕ*_*ij*_(*R*_*ij*_) is the pair potential which is a function of the distance *R* between atoms *i* and *j*. The *ρ*_*h,i*_ represents the host electron density at atom *i* due to the remaining atoms of the system which is approximated by the superposition of atomic densities as follows,

2 $${\rho }_{h,i}=\sum _{j(\ne i)}{\rho }_{j}^{a}({R}_{ij})$$

where ${\rho }_{j}^{a}(R)$ is the electron density at the site of atom *i* due to the presence of atom *j* at a distance of *R*.

The potential functions in Eqs (1) and (2) are usually treated as some fitting functions were proposed by some researchers in consideration of the physical properties of the interested metals as well as their alloys. Generally, the EAM potential is simple; however its embedded energy and pair potential are given in the form of spline functions which leads to some inconvenience for calculations^14^. This explains why there are so many EAM based interatomic potentials developed or optimized in the literature. The reason for evaluating multiple EAM potentials is that, it is a widely used semi-empirical potential formalism for metals which is rooted in the density-functional theory^15^. Over the past decade, several many-body potential models have been designed, which many of those originated from quantum mechanics and share similar mathematical forms with the EAM, to name a few, the Finish-Sinclair model^16,17^ second-order moment approximation of tight-binding^18^, and the effective medium-theory model^19^.

Evaluation of interatomic potentials for three popular metals in nanoscience and technology, platinum gold and silver, on elastic properties is proceeded in this section. The potentials namely *Pt.lammps.eam, Au.lammps.eam, Ag.lammps.eam* were generated by Sheng *et al*.^12,20^ and the rest were obtained from NIST IPR^3^ which were fitted based on their original references. Before tensile strain, the simulation box was relaxed using two equilibration steps. The equilibration step allows the lattice to expand to a desired temperature with a pressure of zero bar at each simulation cell boundary. In all equilibration stages, linear momentum was zeroed by subtracting the center-of-mass velocity of the group from each atom. To calculate the anisotropic elastic stiffness constants, C_11_ and C_12_, a uniaxial tensile strain rate of 10^−3^ ps^−1^ (strain increases 0.1% every picosecond) was applied along the [100] (x- direction) for each studied temperate which leads to nonzero stress components *σ*_*xx*_, *σ*_*yy*_, *σ*_*zz*_^21^. The strains in the y- and z- directions were both controlled to be zero under the NVT ensemble to find the two stiffness constants C_11_ and C_12_ having *ε*_*yy*_ = *ε*_*zz*_ = 0. Once the stress-strain curves are obtained, it is straightforward to find the elastic constants from the slope of the linear part of stress versus strain curves. The correlations and related equations can be found in Supplementary Information.

To find C_44_ using MD simulation by the LAMMPS, a prism region was created to define a triclinic simulation box with initial tilt factors of zero. Then, with the same equilibration steps and under the NVT ensemble, the system was distorted in the [110] direction by applying an engineering shear strain rate of 10^−3^ ps^−1^ so that the crystal is no longer cuboidal. Afterward, C_44_ has been calculated by finding the slope of linear portion of yield curve for *σ*_*xy*_ versus the associated strain *e*_*xy*_ according to (10). In linear regressions applied here, the *R*^2^ coefficient of determination was selected greater than 0.999 to achieve an accurate judgment on the results and having exact slopes as stiffness constants.

Once the elastic constants using different potentials are found, the anisotropic single-crystal elastic constants can be converted into isotropic polycrystalline elastic moduli using the Viogt-Reuss-Hill approximation^22^ which is an averaging scheme. For a single-phase crystalline aggregate made of crystals that are slightly anisotropic, the approximation gives the realistic values of isotropic elastic moduli^23^. This approach combines the upper and lower bounds by assuming the average of values obtained through the Voigt^24^ and Reuss^25^ averaging methods. In the upper bound (Voigt) the strain assumes to be uniform and continuous whereas the stresses are allowed to be discontinuous. In the lower bound (Reuss) the stresses are assumed to be continuous and the strains can be discontinuous (see Supplemental Information).

**Figure 1:**
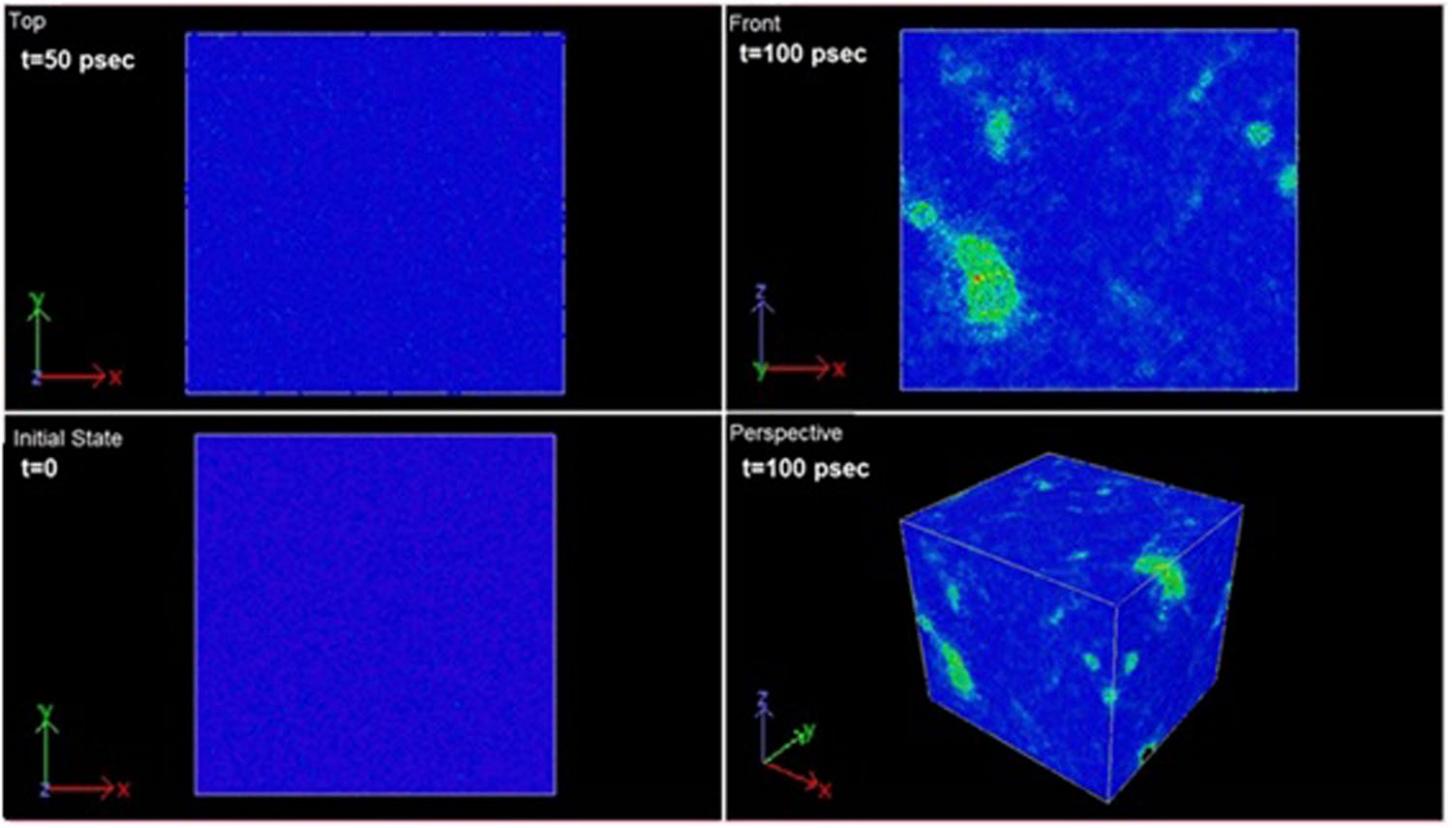
Demonstration of stress per-atom during uniaxial tensile strain evolution.

Figure 1 depicts the strain evolution for the uniaxial tensile visualized by OVITO^26^. The figure shows contour images at different time of strain, visualized by colour coding according to computed stress per-atom by LAMMPS. Tensile strain of 500,000 atoms single crystal of gold at 300 K loaded in the [100] as an instance. As expected, stress on each atom increases by time until the nano-sized single crystal cannot tolerate the stress and necking begins after the ultimate strength is reached. During necking, the material can no longer withstand the maximum stress and the strain increases in the specimen rapidly. stress per-atom is shown for shear strain in Supplemental Fig. S1.

The Common Neighbor Analysis (CNA) algorithm is designed to compute a fingerprint for pairs of atoms, which is designed to characterize the local structural environment for the system^27^. This is an effective filtering method to categorize atoms in crystalline systems to get an accurate understanding of which atoms are associated with which phases, and which are associated with defects^28^. The lattice deformation results in the random formation of FCC and HCP domains, with dislocations at the domain boundaries. These dislocations enable atoms to undergo a shift from FCC to HCP sites, or vice versa. These shifts lead to missing atoms, and therefore a later deposited layer can have missing planes compared to a previously deposited layer. This dislocation formation mechanism can create tensile stress in FCC films^29^. The probability that such transformations are formed is shown in Supplemental Fig. S2 for gold single crystal strains. As the strain continues, the shifting from FCC to other types of sites increases especially in the plastic region.

To characterize whether the atom is part of a perfect lattice, a local defect (e.g. a dislocation or stacking fault), or at a surface the centro-symmetry parameter (CSP) is calculated in the code during the strains along [100] and [110]. Once CSP values are computed by LAMMPS, the Color Coding modifier in OVITO is used to color atoms according to their CSP value. The snapshots shown in Supplemental Fig. S3 give a graphical view of the centro-symmetry parameter of each particle visualized by OVITO and computed based on Kelchner *et al*. formula^30^.

## Results

**Figure 2:**
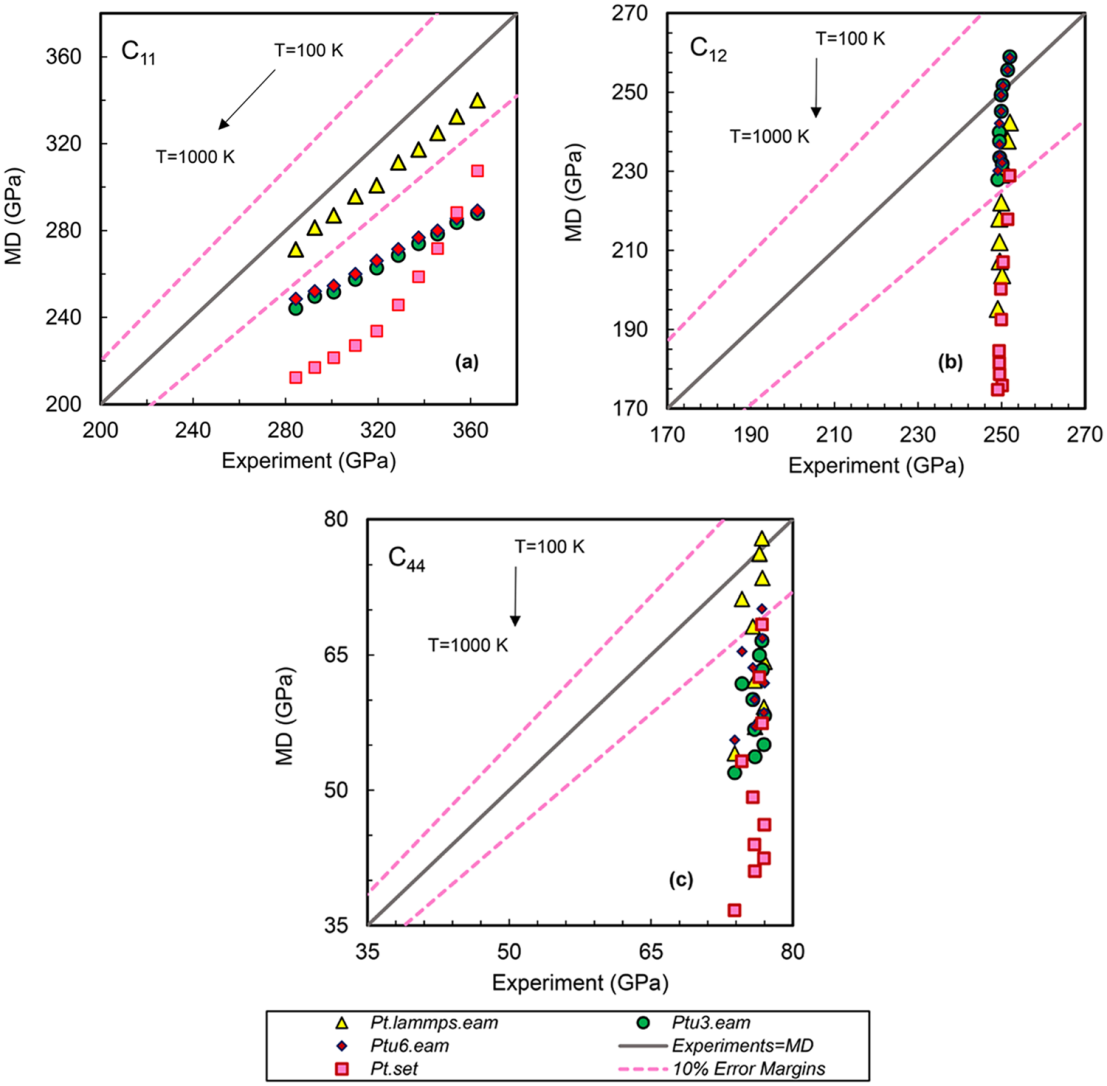
Accuracy of MD results for different Platinum interatomic potentials at various temperatures. (**a**) C_11_, (**b**) C_12_ and (**c**) C_44_.

**Figure 3:**
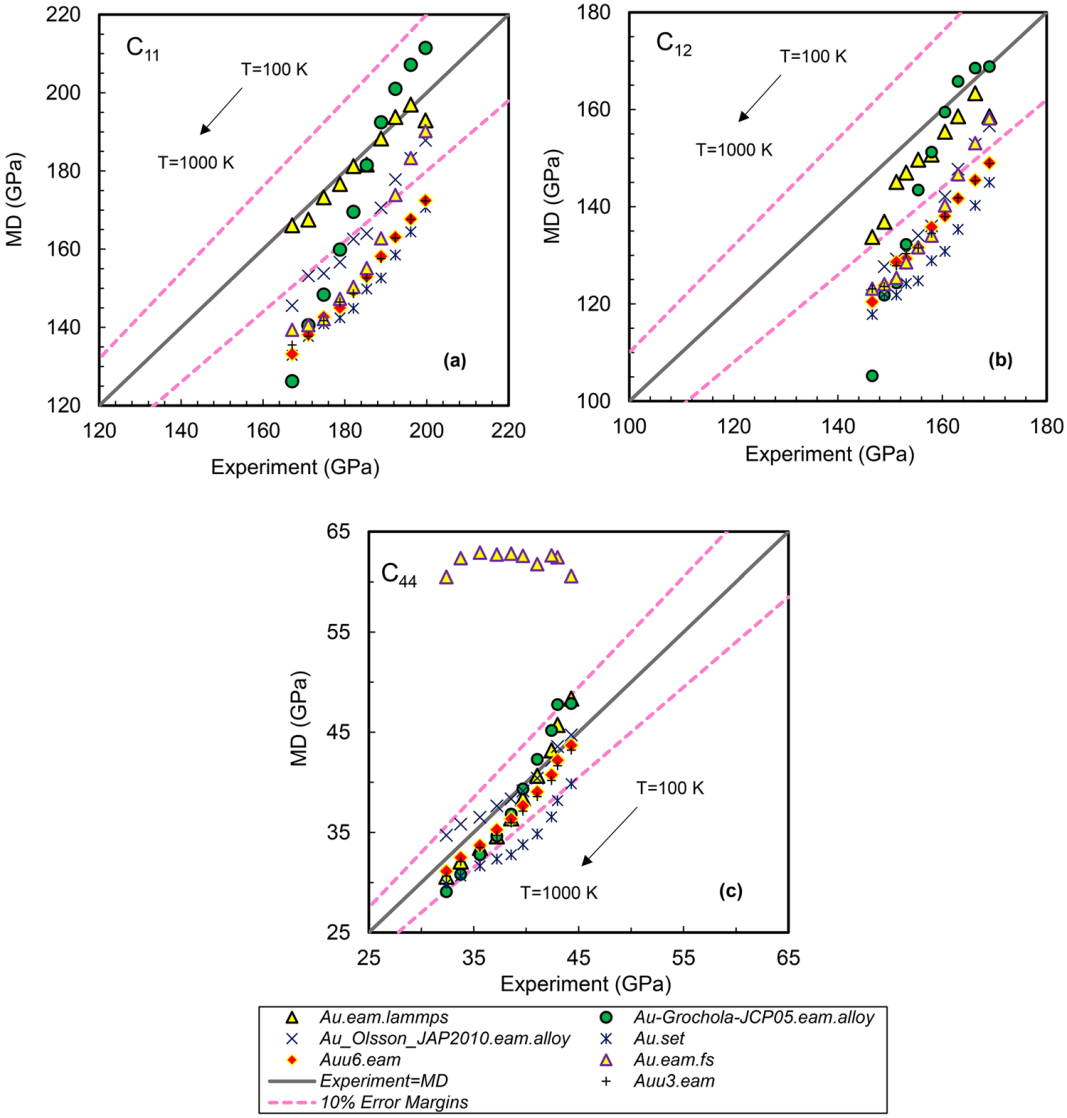
Accuracy of MD results for different Gold interatomic potentials at various temperatures. (**a**) C_11_, (**b**) C_12_ and (**c**) C_44_.

**Figure 4:**
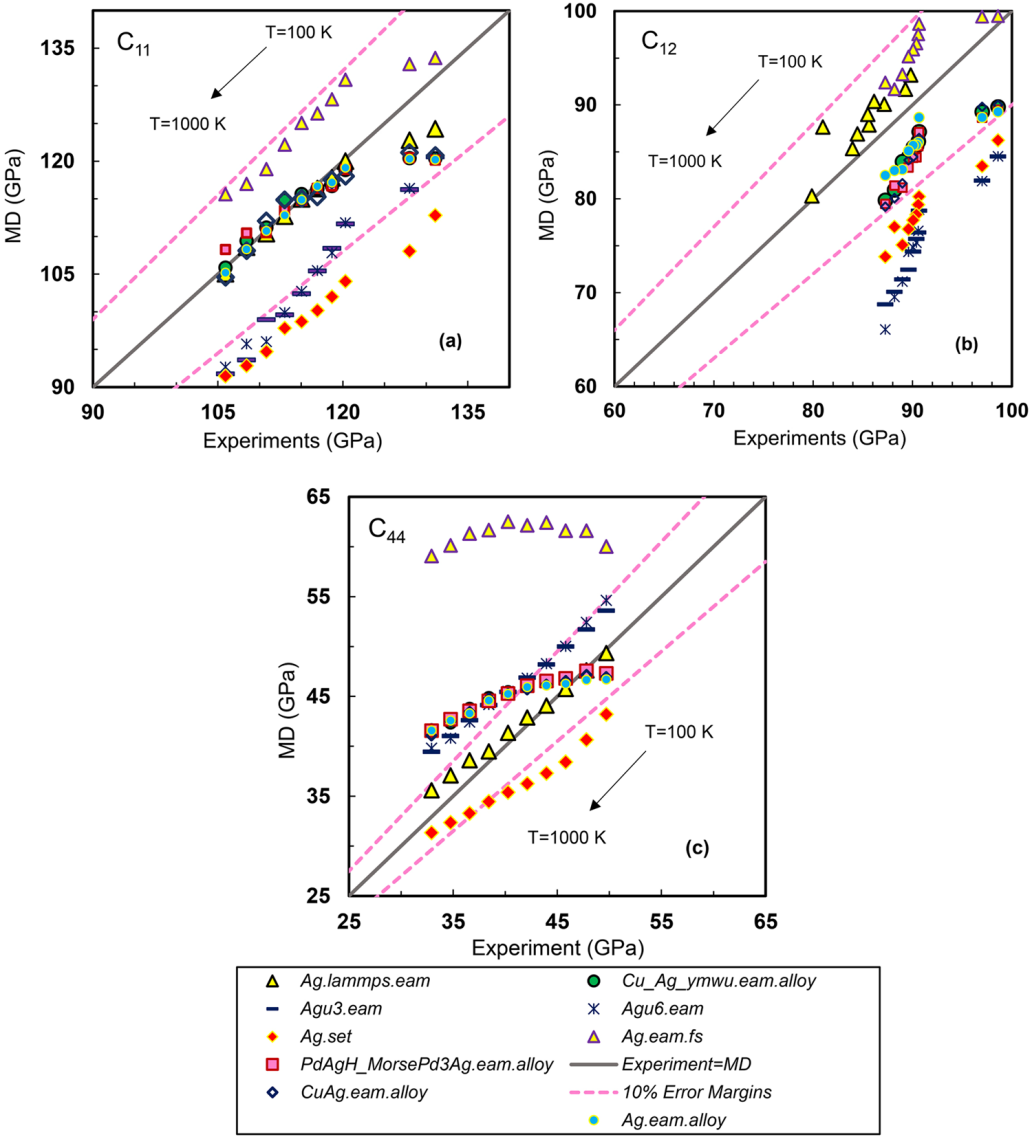
Accuracy of MD results for different Silver interatomic potentials at various temperatures. (**a**) C_11_, (**b**) C_12_ and (**c**) C_44_.

Elastic stiffness constants as one of the leading properties for fitting an interatomic potential have been calculated using a series of widely used EAM based interatomic potentials and are shown in Figs 2–4. In these figures, a straight line has been plotted that starts from minimum MD/experimental value and goes to the maximum possible values of x/y axes at a 45° angle slope to visualize the actual deviation from ideal results. The x-axis shows the experimental elastic constants, while the y-axis shows the constants obtained from MD calculation using each of the force fields. Two border lines are drawn around the ideal line (MD = Experiment) which show that results in this area are within 10% error relative to experimental values. Exact values of relative errors of MD results with respect to experimental results as a function of temperature for all the potentials can be also seen in Supplemental Tables S1 to S20, for Pt, Au and Ag single crystals.

It should be noted that one may find a different relative error with respect to experimental values by using a different criterion, such as using a different R-squared value than the very accurate one that we picked here. The elastic modulus of the studied elements including bulk modulus, shear modulus and Young’s modulus are obtained using VRH method and capability of the interatomic potentials for prediction of these moduli is investigated in Supplemental Figs S4 to S12.

### Platinum Interatomic potentials

Figure 2(a–c) shows the comparison of MD simulation results to experimental ones presented by Collard and McLellan^31^ at different temperatures for four platinum EAM potentials. As can be seen, *Pt.lammps.eam* can predict the C_11_ at all temperatures in excellent agreement with experiments. This is accurate to predict C_12_ and C_44_ but not at temperatures higher than 600 K. This potential was generated by Sheng *et al*.^12,20^ based on *Pt* crystal structures and physical properties which have been validated against experimental results at 0 K and 300 K^12^. The potential was developed by fitting the potential-energy surface (PES) of each element derived from high-precision first-principles calculation by considering a variety of properties of the elements. According to the figure *Ptu3.eam* predicts the C_12_ more accurate than C_11_ and C_44_ in all studied temperatures. This model may not be appropriate for use at high temperatures as its validity already has been approved at 0 K. The potential *Ptu6.eam* yields accurate C_44_ from 100 K to 300 K, while its results are not within 10% error limit. Choice of this force field depends on the users’ criteria and expected accuracy. Since the potentials *Ptu6.eam* and *Ptu3.eam* have the same nature and were created based on a similar method and properties^32^, the results are similar for both interatomic potentials; while *Ptu6.eam* can predict C_12_ at all studied temperatures within 8% error. An acceptable error for three independent elastic constants can be seen at 100 K and maybe 200 K for *Pt.set*. The average error of 20% is seen for MD results obtained by *Pt.set* for predicting elastic stiffness constants from 300 to 1000 K for all the constants. This potential does not seem to be appropriate for temperatures higher than 300 K. The bulk, shear and Young modulus are compared with experimental results in Supplemental Figs S4 to S6 for platinum. As seen in Supplemental Fig. S4, the interatomic potentials *Pt.lammps.eam*, *Ptu3.eam*, *Ptu6.eam* yield more accurate results than *Pt.set* at all temperatures for predicting the bulk modulus. Although *Pt.lammps.eam* shows the best capability up to 300 K, but *Ptu3.eam* and *Ptu6.eam* look the bests at higher temperatures for predicting bulk modulus. However, it is very critical to consider the effect of algebraic expressions and multiplications of the two C_11_ and C_12_ in bulk modulus formula that compensates the errors of the two variables stochastically. Therefore, it is recommended the users implement the *Pt.lammps.eam* since it predicts all three constants at all temperatures with less error with respect to the other ones. Since *Pt.lammps.eam* is the most accurate one in predicting C_44_, it predicts the shear and Young’s modulus better than the other close to experimental results per Supplemental Figs S5 to S6. This force field is recommended to users for obtaining elastic modulus at all the temperatures since it is the closest one to experiments with respect to the other three EAM interatomic potentials.

### Gold Interatomic potentials

Accuracy of gold interatomic potentials versus temperature has been shown in Fig. 3(a–c) and Supplemental Tables S5 to S11. According to the figure, the potential *Au.eam.lammps* can predict all the constants accurately and can be deemed as a completely transferable one for gold. The relative errors with respect to experiments^33^ are low and acceptable from 100 to 1000 K using this potential. This force field predicts all the modulus for axial tensile and in a shear strain across a face, with least errors at all the studied temperatures.

As Fig. 3 shows, the potential *Au-Grochola-JCP05.eam.alloy* that is generated by Becker^3^, based on EAM fitting presented by Grochola *et al*.^34^, showed a good overall agreement with the experimental elastic constants from 100 to 700 K. It is accurate at all temperatures for C_44_, while it reproduces C_11_ and C_12_ with higher relative errors from 800 to 1000 K. This gold EAM potential has been generated using an improved force matching methodology which included fitting to high-temperature solid lattice constants and liquid densities. According to the figure, ability of *Au_Olsson_JAP2010.eam.alloy*^35^ for prediction of elastic constants, decreases by increasing temperature notwithstanding it is yielding accurate results with acceptable relative errors. This potential can be a good candidate in the studied range. This force field is more accurate in predicting C_44_ than C_11_ and C_12_ in temperatures higher than 400 K. Relative errors for C_11_ and C_12_ is less than 10% in that range and goes up to 12.91% at 1000 k for C_11_ and increases from 13.86% at 500 K to 16.97% at 1000 K for C_12_. It also finds the C_44_ with less than 3% error from 100 K to 800 K and less than 8% at 900 to 1000 K.

The interatomic potential *Au.set* which is generated based on Zhou *et al*.^29^ does not show a suitability for producing accurate results from 100 to 1000 K. This potential may not be recommended for this temperature range since it predicts elastic stiffness constants with much higher errors in comparison with experiments.

Results obtained from *Auu3.eam*^32^ and *Auu6.eam*^32^ yield very precise C_44_ with less than 7% error while they predict C_11_ and C_12_ with more than 10% deviation from experiments at 100 K, to around 20% at 1000 K.

The potential *Au.eam.fs* which is converted from Ackland’s^36^ N-body potential was constructed using the approach of Finnis and Sinclair^15,16^. The total energy in this potential is regarded as consisting of a pair-potential part and a many body cohesive part. It is clear from the Fig. 3 that it fails to work for predicting of gold C_44_ in the studied range and is appropriate only for finding C_11_ and C_12_ especially at temperatures below 400 K.

Supplemental Fig. S7 demonstrates the achievement of gold interatomic potentials in finding bulk modulus in which accuracy of predicted C_11_ and C_12_ are important for calculating bulk modulus. It is evident from the figure that *Au.lammps.eam* successfully predicts gold bulk modulus close to experimental results with respect to other potentials. The force field *Au-Grochola-JCP05.eam.alloy* is also accurate for finding bulk modulus up to 600 K and much less for higher temperatures.

For calculating Young’s and shear modulus using VRH model, C_44_ plays a key role besides C_11_ and C_12_. Hereupon, per Supplemental Figs S8 and S9, *Auu3.eam* and *Auu6.eam* as well as *Au.set* which predict C_44_ more accurate than C_11_ and C_12_, are yielding acceptable results up to 300 K. Therefore, the potentials which predict C_44_ precisely such as *Au.lammps.eam*, *Au-Grochola-JCP05.eam.alloy* and *Au_Olsson_JAP2010.eam.alloy* are matched well with experimental values with reasonable relative errors which confirm they are accurate for computing gold shear and Young’s modulus. It can be said that *Au-Grochola-JCP05.eam.alloy* is the most accurate interatomic potential than the rest for predicting gold shear and Young’s modulus from 100 to 1000 K.

### Silver Interatomic potentials

As can be seen in Fig. 4(a–c) and Supplemental Tables S12 and S20, the potential *Ag.lammps.eam* demonstrates robustness at all the studied temperatures for predicting silver elastic properties. This potential can reproduce the C_11_ with less than 5% at 100 and 200 K while predicts this stiffness constant with less than 1% error at higher temperatures. The potential is also accurate for C_12_ with less than 5% deviation from the empirical ones measured by Wolfenden and Harmouche^37^. The constant C_44_ also has been predicted successfully with less than 2% error from 100 to 500 K, within 3% for 600 to 700 K and within 9% for higher temperatures.

It can be found from Fig. 4 that *Cu_Ag_ymwu.eam.alloy* is also an appropriate choice for having right results for C_11_ and C_12_ from 100 to 1000 K. However, it shows competency for prediction of C_44_ only from 100 to 500 K and fails to give appropriate results above that temperature. This potentials is provided by Wu^38^ in which a binary EAM potential is optimized for Cu on Ag(1 1 1) by fitting to ab initio data.

The two potentials *Agu6.eam* and *Agu3.eam* are yielding results close to each other (see Supplemental Tables S14 and 15). They both show ability of reproducing C_11_ and C_44_ within 10% relative error until 400 K while the C_12_ has been calculated with about 15% error in this range and this error increases to 25% at 1000 K. *Ag.eam.alloy* has been generated for Ag by fitting to experimental and the first-principles data. The potential accurately reproduces the lattice parameter, cohesive energy, elastic constants, phonon frequencies, thermal expansion, lattice-defect energies, as well as energies of alternate structures of Ag based on William *et al*.^39^. This potential accurately reproduces C_11_ and C_12_ at all temperatures while it works great only for C_44_ from 100 to 500 K. These imperfections are normal since the potential is fitted to a limited number of experimental data, and may not be adequate to describe phase spaces where the potential was not trained.

According to Fig. 4 *Ag.set* may not be a safe choice for silver elastic constants predictivity since it predicts elastic constants with high discrepancies with experimental ones (more than 10%). This potential has been generated based on the method presented by Zhou *et al*.^29^ in which MD simulated atomic configuration of a (10 Å) compound multilayer deposited on a *Cu* substrate at a temperature of 300 K.

From Fig. 4, it is clear how precisely *Ag.eam.fs* can predict constants for uniaxial tensile while it is inaccurate for C_44_. In Supplemental Table S19 and Fig. 4, MD results using *PdAgH_MorsePd3Ag.eam.alloy* interatomic potential are reported. It is evident from the figure that this EAM potential is able to precisely predict C_11_ and C_12_ from 100 to 1000 K also for C_44_ from 100 to 600 K, however may not be able to reproduce the C_44_ very accurate.

To explore the exactitude of the interatomic potentials on silver elastic modulus, Supplemental Figs S10 to S12 compare the bulk, shear and Young’s modulus correspondingly at the designated temperatures. Supplemental Fig. S10 shows the ability of the EAM potentials in producing silver bulk modulus with acceptable agreements with experiments except for those of *Agu3.eam*, *Agu6.eam* and *Ag.set* which do not adequately reproduce the C_11_ and C_12_, the necessary constants for bulk modulus calculation. The scenario changes for shear and Young’s modulus and it is clear from Supplemental Figs S11 and S12 that some of the potentials that are successful in producing bulk modulus, now fail to predict accurate shear and Young’s modulus at some temperatures. This is because they could not appropriately predict C_44_. On the other hand, it can be seen from the figures that potentials which were not able to produce none of the constants with small relative errors, are close to the experiments. This is deceptive result that may mislead users to choose an inappropriate force field for their atomistic simulation. Such stochastic agreements are due to arithmetical calculations of the three constants whose product might be equal to the experimental ones by chance. Hence, it is recommended that users employ the interatomic potentials which produce both elastic constants and elastic modulus precisely. So, based on Fig. 4 and Supplemental Figs S11 and S12, for producing silver shear and Young’s modulus, the interatomic potential *Ag.lammps.eam* is suggested for all designated temperatures and *PdAgH_MorsePd3Ag.eam.alloy*, *CuAg.eam.alloy*, *Ag.eam.alloy*, and *Cu_Ag_ymwu.eam.alloy* are recommended for 100 to 600 K since they are in acceptable agreemens with experimental results.

## Summary and Conclusions

Transferability of several interatomic potentials for high-accuracy atomistic computer simulations is considered to introduce accurate ones for solving a wider range of scientific and engineering challenges. Investigations were undertaken using MD simulations from 100 to 1000 K for three precious transition metals popular in nanoscience and nanotechnology: platinum, gold and silver. Elastic properties, one of the main standards and principles for the fitting process of an interatomic potential, were used as benchmarks. We have shown which potentials are effective and applicable at each temperature. In the fitting process, as some specific crystal structures are applied, it is vital to evaluate how well the same force fields work for other crystal structures. For this reason, effect of temperature as one of the leading influential properties have been investigated to evaluate the reliability of potentials at temperatures different to what they have been fitted. Most commonly fitted properties for bulk solid-state materials are bulk energetics, defects, and mechanical properties which are obtained from experiments, when available, or from calculations using quantum mechanics such as density functional theory (DFT) simulations. Fitting properties is determinative for potentials performance. For instance, potentials fit to experimental elastic constant data will probably better reproduce the elastic constants. Fitting methodology also plays a main role in predictivity of interatomic potentials.

As shown Figs 2(a–c) to 4(a–c) in results section, supplemental Figs S4–S12 and supplemental Tables S1–S20, interatomic potentials generated by Sheng *et al*.^12^ are very accurate with wide applicability to various bulk constants of platinum, gold and silver. The potentials *Pt.lammps.eam*, *Au.lammps.eam* and *Au.lammps.eam* were developed by fitting the potential-energy surface (PES) of each element derived from high-precision first-principles calculations. This is so important since thermodynamics and kinetics of materials are dictated by their PESs. The improved accuracy of their EAM potentials is due to the method they applied to develop the potentials. During the potential development, the EAM potentials were fit to the ab initio databases that adequately describe the potential energy landscapes of the metallic systems. For instance, extensive ab initio molecular dynamics simulations were conducted to obtain the atomic trajectories of systems (e.g., fcc and hcp) along the melting sequence. The forces and tensors of selected atomic configurations at different temperatures were incorporated into the ab initio database for potential fitting. In other words, the potentials were developed to match ab initio MD results which were slightly corrected with experimental inputs. While they followed the same fitting procedures in developing the potentials for various elements, the potentials were optimized to have the best overall performances. By saying that, they refer to the capability of the potentials to describe many different properties (mechanical, thermal, liquids, defects, etc.) with reasonable accuracy. Since different elements have different properties (due to their different electronic configurations), the accuracy of the EAM potential in describing all the properties may not be equal. For platinum, it is possible that high-temperature thermal expansion as predicted by the EAM may not be as accurate as that at low temperatures. This is why we see accurate results for *Au.lammps.eam* and *Au.lammps.eam* from 100 K to 1000 K, but *Pt.lammps.eam* reproduces acceptable results up to 600 K. This is the same for potentials generated based on fitting and parametrization presented by Foiles *et al*.^40^ (*Ptu3.eam*, *Auu3.eam* and *Agu3.eam*) and Adams *et al*.^32^ (*Ptu6.eam*, *Auu6.eam* and *Agu6.eam*). They were all fitted to pure metal properties used to determine the functions: equilibrium lattice constants, sublimation energy, bulk modulus, elastic constants, and vacancy-formation energy. However, they found their calculated results based on these EAM models for platinum show discrepancies for all elastic constants much higher (up to 8 times) than those of calculated ones for gold and silver. Therefore, we see these potentials yield more acceptable results for gold and silver than those of platinum. Foiles *et al*.^40^ found that C_11_ calculated for platinum has higher error than C_12_ and C_44_ which confirms what we presented in this work.

Potentials that have been generated based on Zhou *et al*.^29^ *Pt.set, Au.set* and *Ag.set* are not reproducing results with high accuracy at different temperatures since they were designed by specifically fitting the parameters of the EAM potentials to alloy properties (such as the heat of solution). Hence, as they have been devised for some alloys, may not be so accurate for single elements for this reason.

The reason that *Au-Grochola-JCP05.eam.alloy*^34^ successfully predicts gold elastic constants in a wide temperature range, is fitting their EAM potential using an improved force matching methodology which involves the use of scaled liquid ab initio force data to fix the potential pair repulsive core and brute force fitting to high-temperature solid lattice constants and liquid densities. Thermal expansion for this potential has good agreement to experimental solid lattice constants especially from 0 to 1000 K due to the fitting of these values. The methodology they applied, produces a potential with good overall agreement to a range of properties including elastic constants which is desired here.

*Au_Olsson_JAP2010.eam.alloy*^35^ was fitted exactly to the second order elastic constants at 0 K and by evaluating the third order elastic constants, it is revealed that it predicts results in reasonable agreement with experimental as well as ab initio. This EAM potential captures linear and non-linear elastic properties of the bulk very well. The cell thermal expansion is fitted to a cubic polynomial of the temperature between 0 K and 700 K.

Hale^41^ used the Morse-style function (as opposed to the Hybrid) for the Pd-Ag interaction. In the fitting process of this potential, transformation constant set to the unique value which results in both the embedding and pair functions being independently minimized for the ideal FCC structure and lattice spacing. Since they directly used explicit forms of Williams^39^ potential for Ag, and this potential provides excellent structural, lattice and elastic properties, subsequently *PdAgH_MorsePd3Ag.eam.alloy* is reasonable for silver elastic properties calculations at different temperatures.

William *et al*.^39^, except for thermal expansion factors of pure Ag, fitted all target properties used in the potential refer to 0 K. Their deliberately chosen strategy aimed to increase the transferability of the potential to high-temperature properties. The potentials they fitted successfully demonstrated good transferability to high-temperature properties. For this reason, both *CuAg.eam.alloy* and *Ag.eam.alloy* which are generated according to William *et al*.^39^, are transferable for silver elastic properties calculation.

However, potentials developed and optimized by Sheng *et al*.^12^ excel over the rest of studied potentials because they predict larger planar defect energies that are more in line with experimental values and DFT predictions.

This study diagnoses inevitable shortcomings from potentials that were developed and trained to a limited number of experimental data. The present study also shines a spotlight on the interatomic potentials that demonstrate good transferability to finite temperature properties and can be safely used for advancing nanoscale breakthroughs. This work may be useful for others who intend to employ these or other semi-empirical potentials as it provides an organized framework, and may preclude incorrect simulation results in studies of the properties of clusters and/or crystals of the same materials. It will also make research faster by readily providing a thorough examination and compilation of the performance of several interatomic potentials.

## Electronic supplementary material

Supplemental information
