# Supplementary material for: Interatomic Potentials Transferability for Molecular Simulations: A Comparative Study for Platinum, Gold and Silver
Source: Sci Rep. 2018 Feb 5;8:2424. doi: 10.1038/s41598-018-20375-4 (PMC5799210; doi:10.1038/s41598-018-20375-4)
Supplement: Supplementary file 1 — Supplemental information [file 41598_2018_20375_MOESM1_ESM.pdf]

Supplemental information for:

Interatomic Potentials Transferability for Molecular Simulations: A Comparative Study for Platinum, Gold and Silver

Seyed Moein Rassoulinejad-Mousavi **and** Yuwen Zhang

Department of Mechanical and Aerospace Engineering, University of Missouri, Columbia, Missouri 65211, USA

Email: zhangyu@missouri.edu

The way that elastic constants are obtained[1]:

Elastic constants C relate the strain ε and the stress σ in a linear fashion:

$$\sigma_{ij} = \sum_{kl} C_{ijkl}\varepsilon_{kl} \tag{1}$$

which relates to elastic energy density U that is defined as follows [22] for a cubic crystal x- along [100], y || [010], z || [001] :

$$U = \frac{1}{2}C_{11}(e_{xx}^2 + e_{yy}^2 + e_{zz}^2) + \frac{1}{2}C_{44}(e_{yz}^2 + e_{zx}^2 + e_{xy}^2) + C_{12}(e_{yy}e_{zz} + e_{zz}e_{xx} + e_{xx}e_{yy}), \tag{2}$$

where e_{ij} are strain components by the relations:

$$\begin{aligned} &e_{xx} \equiv \varepsilon_{xx} = \frac{\partial u}{\partial x}; \qquad e_{yy} \equiv \varepsilon_{yy} = \frac{\partial v}{\partial y}; \qquad e_{xx} \equiv \varepsilon_{zz} = \frac{\partial w}{\partial z}, \\ &e_{xy} \equiv \varepsilon_{yx} + \varepsilon_{xy} = \frac{\partial u}{\partial y} + \frac{\partial v}{\partial x}; \quad e_{yz} \equiv \varepsilon_{zy} + \varepsilon_{yz} = \frac{\partial v}{\partial z} + \frac{\partial w}{\partial y}; \quad e_{yz} \equiv \varepsilon_{zy} + \varepsilon_{yz} = \frac{\partial u}{\partial z} + \frac{\partial w}{\partial x}; \end{aligned} \tag{3}$$

where u, v and w are displacements in x-, y- and z- directions.

[1] *C. Kittel, Introduction to solid state physics, 8th ed. (Wiley, 2005).*

Having:

$$\frac{\partial U}{\partial e_{ij}} = \sigma_{ij} \tag{4}$$

Then, we derive the following relations according to (3) to (6):

$$\sigma_{xx} = C_{11}e_{xx} + C_{12}(e_{yy} + e_{zz}), \tag{5}$$

$$\sigma_{yy} = C_{11}e_{yy} + C_{12}(e_{xx} + e_{zz}), \tag{6}$$

$$\sigma_{zz} = C_{11}e_{zz} + C_{12}(e_{xx} + e_{yy}), \tag{7}$$

$$\sigma_{xy} = C_{44}e_{xy}. \tag{8}$$

where, e_{xy} is also known as γ_{xy}, which is the engineering shear strain.

Hence C_{11}, C_{12} and C_{44} can be find from the linear portion of σ_{xx} versus e_{xx}, σ_{yy} versus e_{xx} and σ_{xy} versus e_{xy}, respectively.

The Voigt-Reuss-Hill Approximation[2]:

Bulk modulus is defined similarly under all the three approximations and can be exactly defined as follows,

$$B_{VRH} = B_V = B_R = \frac{C_{11} + 2C_{12}}{3}, \tag{9}$$

and the upper and lower bounds of shear moduli are,

[2] *R. Hill, Proc. Phys. Soc. 65, 349 (1952).*

$$G_V = \frac{C_{11} - C_{12} + 3C_{44}}{5}, \qquad (10)$$

and

$$G_R = \frac{5C_{44}(C_{11} - C_{12})}{4C_{44} + 3(C_{11} - C_{12})}, \qquad (11)$$

Knowing shear modulus *G* and bulk modulus *B*, it is then straightforward to find Young's modulus *E* from the well-known isotropic relations as follows,

$$E = \frac{9GB}{3B + G} \ , \qquad (12)$$

Tables S1 to S20

Table S1 Predicted platinum elastic stiffness constants by the interatomic potential *Pt.lammps.eam*

**Pt.lammps.eam**									
T (K)	C_{11} (GPa)	C_{11} (Expt.)	Error (%)	C_{12} (GPa)	C_{12} (Expt.)	Error (%)	C_{44} (GPa)	C_{44} (Expt.)	Error (%)
100	340	363	6.33	242.32	252	3.84	77.91	76.74	1.52
200	332.46	354	6.08	237.76	251.5	5.46	76.211	76.47	0.34
300	324.92	345.75	6.02	231.26	250.4	7.64	73.52	76.77	4.23
400	317.35	337.54	5.98	229.18	249.9	8.29	71.2	74.62	4.58
500	311.39	328.78	5.29	222.1	249.96	11.15	68.15	75.78	10.07
600	300.85	319.46	5.83	218.03	249.46	12.60	64.24	77	16.57
700	295.72	310.15	4.65	212.06	249.52	15.01	62.15	75.95	18.17
800	287.01	300.83	4.59	207.21	249.57	16.97	59.24	76.95	23.01
900	281.38	292.62	3.84	203.69	250.17	18.58	57	76	25.00
1000	271.37	284.41	4.58	195.17	249.13	21.66	54.08	73.85	26.77

Table S2 Predicted platinum elastic stiffness constants by the interatomic potential *Ptu3.eam*

Ptu3.eam									
T (K)	C_{11} (GPa)	C_{11} (Expt.)	Error (%)	C_{12} (GPa)	C_{12} (Expt.)	Error (%)	C_{44} (GPa)	C_{44} (Expt.)	Error (%)
100	287.95	363	20.67	258.96	252	2.68	66.56	76.74	13.27
200	283.70	354	19.85	255.59	251.5	1.62	64.98	76.47	15.03
300	278.41	345.75	19.48	251.68	250.40	0.51	63.34	76.77	17.49
400	273.97	337.54	18.83	249.33	249.90	0.23	61.81	74.62	17.17
500	268.51	328.78	18.33	245.16	249.96	1.92	60.07	75.78	20.73
600	262.71	319.46	17.76	239.90	249.46	3.83	58.28	77.00	24.31
700	257.52	310.15	16.97	237.61	249.52	4.77	56.75	75.95	25.28
800	251.71	300.83	16.33	233.47	249.57	6.45	55.05	76.95	28.46
900	249.67	292.62	14.68	231.87	250.17	7.32	53.72	76.00	29.32
1000	244.18	284.41	14.15	227.92	249.13	8.51	51.95	73.85	29.65

Table S3 Predicted platinum elastic stiffness constants by the interatomic potential *Ptu6.eam*

**Ptu6.eam**									
T (K)	C_{11} (GPa)	C_{11} (Expt.)	Error (%)	C_{12} (GPa)	C_{12} (Expt.)	Error (%)	C_{44} (GPa)	C_{44} (Expt.)	Error (%)
100	289.24	363	20.31	258.76	252	2.61	70.13	76.74	8.61
200	285.62	354	19.31	255.63	251.5	1.61	68.58	76.47	10.32
300	279.97	345.75	19.03	251.63	250.40	0.49	66.87	76.77	12.90
400	276.74	337.54	18.01	249.28	249.90	0.25	65.38	74.62	12.38
500	271.45	328.78	17.44	245.21	249.96	1.90	63.60	75.78	16.07
600	266.12	319.46	16.70	242.13	249.46	2.94	61.87	77.00	19.65
700	259.97	310.15	16.18	236.83	249.52	5.09	60.04	75.95	20.95
800	254.54	300.83	15.39	233.73	249.57	6.35	58.61	76.95	23.83
900	252.04	292.62	13.87	232.14	250.17	7.21	57.06	76.00	24.92
1000	248.52	284.41	12.62	230.12	249.13	7.63	55.57	73.85	24.75

Table S4 Predicted platinum elastic stiffness constants by the interatomic potential *Pt.set*

**Pt.set**									
T (K)	C_{11} (GPa)	C_{11} (Expt.)	Error (%)	C_{12} (GPa)	C_{12} (Expt.)	Error (%)	C_{44} (GPa)	C_{44} (Expt.)	Error (%)
100	307.50	363	15.28	228.90	252	9.16	68.37	76.74	10.91
200	288.31	354	18.55	217.83	251.5	13.38	62.52	76.47	18.24
300	271.75	345.75	21.40	207.03	250.40	17.32	57.42	76.77	25.21
400	258.68	337.54	23.36	200.22	249.90	19.88	53.21	74.62	28.69
500	245.76	328.78	25.25	192.50	249.96	22.99	49.20	75.78	35.08
600	233.70	319.46	26.85	184.54	249.46	26.02	46.17	77.00	40.04
700	227.06	310.15	26.79	181.48	249.52	27.27	43.95	75.95	42.13
800	221.46	300.83	26.38	178.67	249.57	28.41	42.45	76.95	44.83
900	216.92	292.62	25.87	175.78	250.17	29.74	41.01	76.00	46.04
1000	212.30	284.41	25.35	174.73	249.13	29.86	36.67	73.85	50.35

Table S5 Predicted gold elastic stiffness constants by the interatomic potential *Au.eam.lammps*

**Au.eam.lammps**									
T (K)	C_{11} (GPa)	C_{11} (Expt.)	Error (%)	C_{12} (GPa)	C_{12} (Expt.)	Error (%)	C_{44} (GPa)	C_{44} (Expt.)	Error (%)
100	192.96	199.70	3.38	158.59	169.00	6.16	48.37	44.30	9.19
200	196.96	196.10	0.44	163.34	166.30	1.78	45.73	43.00	6.35
300	193.80	192.34	0.76	158.60	163.02	2.71	43.20	42.42	1.84
400	188.33	188.84	0.27	155.44	160.47	3.13	40.65	41.05	0.97
500	181.64	185.34	2.00	150.74	157.92	4.55	38.38	39.69	3.30
600	181.22	182.08	0.47	149.69	155.36	3.65	36.40	38.56	5.60
700	176.66	178.81	1.20	147.01	153.05	3.95	34.60	37.20	6.99
800	173.25	174.84	0.91	145.11	151.20	4.03	33.45	35.60	6.04
900	167.57	171.11	2.07	136.92	148.89	8.04	32.08	33.76	4.98
1000	166.11	167.13	0.61	133.80	146.57	8.71	30.58	32.40	5.62

Table S6 Predicted gold elastic stiffness constants by the interatomic potential *Au-Grochola-JCP05.eam.alloy*

**Au-Grochola-JCP05.eam.alloy**									
T (K)	C_{11} (GPa)	C_{11} (Expt.)	Error (%)	C_{12} (GPa)	C_{12} (Expt.)	Error (%)	C_{44} (GPa)	C_{44} (Expt.)	Error (%)
100	211.46	199.70	5.89	168.85	169.00	0.09	47.86	44.30	8.04
200	207.16	196.10	5.64	168.54	166.30	1.35	47.77	43.00	11.09
300	201.04	192.34	4.52	165.82	163.02	1.72	45.18	42.42	6.51
400	192.46	188.84	1.92	159.50	160.47	0.60	42.29	41.05	3.02
500	181.44	185.34	2.10	151.27	157.92	4.21	39.35	39.69	0.86
600	169.55	182.08	6.88	143.45	155.36	7.67	36.83	38.56	4.49
700	159.90	178.81	10.58	132.27	153.05	13.58	34.63	37.20	6.91
800	148.40	174.84	15.12	124.33	151.20	17.77	32.76	35.60	7.98
900	140.55	171.11	17.86	121.78	148.89	18.21	30.80	33.76	8.77
1000	126.24	167.13	24.47	105.19	146.57	28.23	29.08	32.40	10.25

Table S7 Predicted gold elastic stiffness constants by the interatomic potential *Au_Olsson_JAP2010.eam.alloy*

**Au_Olsson_JAP2010.eam.alloy**									
T (K)	C_{11} (GPa)	C_{11} (Expt.)	Error (%)	C_{12} (GPa)	C_{12} (Expt.)	Error (%)	C_{44} (GPa)	C_{44} (Expt.)	Error (%)
100	187.80	199.70	5.96	156.67	169.00	7.30	44.73	44.30	0.97
200	183.24	196.10	6.56	152.85	166.30	8.09	43.58	43.00	1.35
300	177.82	192.34	7.55	147.73	163.02	9.38	42.18	42.42	0.57
400	170.57	188.84	9.67	142.08	160.47	11.46	40.42	41.05	1.53
500	163.98	185.34	11.52	136.03	157.92	13.86	39.14	39.69	1.39
600	162.59	182.08	10.70	134.10	155.36	13.68	38.34	38.56	0.57
700	156.66	178.81	12.39	130.53	153.05	14.71	37.63	37.20	1.16
800	153.84	174.84	12.01	129.24	151.20	14.52	36.50	35.60	2.53
900	153.15	171.11	10.50	127.68	148.89	14.25	35.82	33.76	6.10
1000	145.56	167.13	12.91	121.69	146.57	16.97	34.71	32.40	7.13

Table S8 Predicted gold elastic stiffness constants by the interatomic potential *Au.set*

Au.set									
T (K)	C_{11} (GPa)	C_{11} (Expt.)	Error (%)	C_{12} (GPa)	C_{12} (Expt.)	Error (%)	C_{44} (GPa)	C_{44} (Expt.)	Error (%)
100	170.70	199.70	14.52	145.03	169.00	14.18	39.86	44.30	10.02
200	164.41	196.10	16.16	140.28	166.30	15.65	38.18	43.00	11.21
300	158.51	192.34	17.59	135.33	163.02	16.99	36.54	42.42	13.86
400	152.62	188.84	19.18	130.80	160.47	18.49	34.85	41.05	15.10
500	149.82	185.34	19.16	128.88	157.92	18.39	33.78	39.69	14.89
600	144.85	182.08	20.45	124.76	155.36	19.70	32.78	38.56	14.99
700	142.47	178.81	20.32	124.19	153.05	18.86	32.35	37.20	13.04
800	140.90	174.84	19.41	121.86	151.20	19.40	31.65	35.60	11.10
900	137.72	171.11	19.51	122.07	148.89	18.01	30.66	33.76	9.18
1000	132.88	167.13	20.49	117.82	146.57	19.62	29.88	32.40	7.78

Table S9 Predicted gold elastic stiffness constants by the interatomic potential *Auu3.eam*

**Auu3.eam**									
T (K)	C_{11} (GPa)	C_{11} (Expt.)	Error (%)	C_{12} (GPa)	C_{12} (Expt.)	Error (%)	C_{44} (GPa)	C_{44} (Expt.)	Error (%)
100	172.35	199.70	13.70	148.90	169.00	11.89	43.21	44.30	2.46
200	167.59	196.10	14.54	145.36	166.30	12.59	41.65	43.00	3.14
300	162.89	192.34	15.31	141.85	163.02	12.99	40.19	42.42	5.26
400	157.61	188.84	16.54	137.99	160.47	14.01	38.58	41.05	6.02
500	152.66	185.34	17.63	134.53	157.92	14.81	37.13	39.69	6.45
600	148.67	182.08	18.35	131.53	155.36	15.34	35.95	38.56	6.77
700	146.47	178.81	18.09	130.39	153.05	14.81	34.69	37.20	6.75
800	141.77	174.84	18.91	127.85	151.20	15.44	33.47	35.60	5.98
900	137.75	171.11	19.50	123.59	148.89	16.99	32.08	33.76	4.98
1000	135.52	167.13	18.91	123.09	146.57	16.02	30.63	32.40	5.46

Table S10 Predicted gold elastic stiffness constants by the interatomic potential *Auu6.eam*

**Auu6.eam**									
T (K)	C_{11} (GPa)	C_{11} (Expt.)	Error (%)	C_{12} (GPa)	C_{12} (Expt.)	Error (%)	C_{44} (GPa)	C_{44} (Expt.)	Error (%)
100	172.48	199.70	13.63	149.03	169.00	11.82	43.70	44.30	1.35
200	167.71	196.10	14.48	145.48	166.30	12.52	42.21	43.00	1.84
300	163.09	192.34	15.21	141.70	163.02	13.08	40.75	42.42	3.94
400	158.18	188.84	16.24	138.07	160.47	13.96	39.04	41.05	4.90
500	153.01	185.34	17.44	135.81	157.92	14.00	37.67	39.69	5.09
600	149.36	182.08	17.97	131.66	155.36	15.25	36.35	38.56	5.73
700	145.04	178.81	18.89	129.27	153.05	15.54	35.27	37.20	5.19
800	142.69	174.84	18.39	128.65	151.20	14.91	33.70	35.60	5.34
900	138.12	171.11	19.28	123.78	148.89	16.86	32.50	33.76	3.73
1000	133.20	167.13	20.30	120.46	146.57	17.81	31.12	32.40	3.95

Table S11 Predicted gold elastic stiffness constants by the interatomic potential *Au.eam.fs*

**Au.eam.fs**									
T (K)	C_{11} (GPa)	C_{11} (Expt.)	Error (%)	C_{12} (GPa)	C_{12} (Expt.)	Error (%)	C_{44} (GPa)	C_{44} (Expt.)	Error (%)
100	190.21	199.70	4.75	158.19	169.00	6.40	60.57	44.30	36.73
200	183.27	196.10	6.54	153.06	166.30	7.96	62.43	43.00	45.19
300	173.88	192.34	9.60	146.68	163.02	10.02	62.62	42.42	47.62
400	162.75	188.84	13.82	140.25	160.47	12.60	61.76	41.05	50.45
500	155.18	185.34	16.27	134.06	157.92	15.11	62.59	39.69	57.70
600	150.36	182.08	17.42	131.66	155.36	15.25	62.80	38.56	62.86
700	147.30	178.81	17.62	128.55	153.05	16.01	62.71	37.20	68.58
800	142.11	174.84	18.72	125.35	151.20	17.10	62.91	35.60	76.71
900	140.59	171.11	17.84	124.08	148.89	16.66	62.35	33.76	84.69
1000	139.39	167.13	16.60	123.16	146.57	15.97	60.49	32.40	86.70

Table S12 Predicted silver elastic stiffness constants by the interatomic potential *Ag.lammps.eam*

**Ag.lammps.eam**									
T (K)	C_{11} (GPa)	C_{11} (Expt.)	Error (%)	C_{12} (GPa)	C_{12} (Expt.)	Error (%)	C_{44} (GPa)	C_{44} (Expt.)	Error (%)
100	124.31	131.1	5.18	93.2	89.8	3.79	49.34	49.7	0.72
200	122.8	128	4.06	91.68	89.28	2.69	47.6	47.8	0.42
300	120.02	120.32	0.25	90.08	87.14	3.37	45.74	45.81	0.15
400	117.8	118.69	0.75	90.38	86.13	4.93	44.08	43.96	0.27
500	116.37	116.94	0.49	87.87	85.62	2.63	42.89	42.11	1.85
600	115	115.06	0.05	88.98	85.48	4.09	41.355	40.27	2.69
700	112.72	113.02	0.27	86.91	84.44	2.93	39.49	38.43	2.76
800	110.38	110.82	0.40	85.35	83.96	1.66	38.59	36.59	5.47
900	108.6	108.45	0.14	87.65	80.96	8.26	37.07	34.76	6.65
1000	105.05	105.92	0.82	80.3	79.85	0.56	35.58	32.93	8.05

Table S13 Predicted silver elastic stiffness constants by the interatomic potential *Cu_Ag_ymwu.eam.alloy*

**Cu_Ag_ymwu.eam.alloy**									
T (K)	C_{11} (GPa)	C_{11} (Expt.)	Error (%)	C_{12} (GPa)	C_2 (Expt.)	Error (%)	C_{44} (GPa)	C_{44} (Expt.)	Error (%)
100	120.39	131.1	8.17	89.80	98.6	8.92	47.30	49.7	4.83
200	120.42	128	5.92	89.28	97	7.96	47.60	47.8	0.42
300	118.83	120.32	1.24	87.14	90.64	3.86	46.80	45.81	2.16
400	116.70	118.69	1.68	86.13	90.57	4.90	46.31	43.96	5.35
500	116.35	116.94	0.50	85.62	90.38	5.27	46.02	42.11	9.29
600	115.65	115.06	0.51	85.48	90.05	5.07	45.48	40.27	12.94
700	114.84	113.02	1.61	84.44	89.59	5.75	44.89	38.43	16.81
800	111.20	110.82	0.34	83.96	88.97	5.63	43.82	36.59	19.76
900	109.42	108.45	0.89	80.96	88.18	8.19	42.33	34.76	21.78
1000	105.88	105.92	0.04	79.85	87.24	8.47	41.67	32.93	26.54

Table S14 Predicted silver elastic stiffness constants by the interatomic potential *Agu3.eam*

**Agu3.eam**									
T (K)	C_{11} (GPa)	C_{11} (Expt.)	Error (%)	C_{12} (GPa)	C_{12} (Expt.)	Error (%)	C_{44} (GPa)	C_{44} (Expt.)	Error (%)
100	120.59	131.1	8.02	84.5	98.6	14.30	53.59	49.7	7.83
200	116.27	128.00	9.16	81.93	97	15.54	51.70	47.8	8.16
300	111.71	120.32	7.16	78.71	90.64	13.16	50.00	45.81	9.15
400	108.45	118.69	8.63	76.40	90.57	15.65	48.2	43.96	9.65
500	105.46	116.94	9.82	75.70	90.38	16.24	46.86	42.11	11.28
600	102.44	115.06	10.97	74.36	90.05	17.42	45.45	40.27	12.86
700	99.64	113.02	11.84	72.44	89.59	19.14	44.11	38.43	14.78
800	98.99	110.82	10.67	71.39	88.97	19.76	42.6	36.59	16.43
900	93.65	108.45	13.65	70.07	88.18	20.54	41.03	34.76	18.04
1000	91.78	105.92	13.35	68.73	87.24	21.22	39.44	32.93	19.77

Table S15 Predicted silver elastic stiffness constants by the interatomic potential Agu6.eam

Agu6.eam									
T (K)	C_{11} (GPa)	C_{11} (Expt.)	Error (%)	C_{12} (GPa)	C_{12} (Expt.)	Error (%)	C_{44} (GPa)	C_{44} (Expt.)	Error (%)
100	120.53	131.10	8.06	84.52	98.60	14.28	54.63	49.7	9.92
200	116.4	128.00	9.06	81.89	97.00	15.58	52.41	47.8	9.64
300	111.85	120.32	7.04	78.82	90.64	13.04	50.01	45.81	9.17
400	107.83	118.69	9.15	76.57	90.57	15.46	48.28	43.96	9.83
500	105.49	116.94	9.79	75.31	90.38	16.67	46.83	42.11	11.21
600	102.75	115.06	10.70	74.81	90.05	16.92	45.51	40.27	13.01
700	99.9	113.02	11.61	74.34	89.59	17.02	44.18	38.43	14.96
800	96.06	110.82	13.32	71.19	88.97	19.98	42.44	36.59	15.99
900	95.73	108.45	11.73	69.54	88.18	21.14	40.81	34.76	17.41
1000	92.7	105.92	12.48	66.06	87.24	24.28	39.82	32.93	20.92

Table S16 Predicted silver elastic stiffness constants by the interatomic potential *Ag.eam.alloy*

**Ag.eam.alloy**									
T (K)	C_{11} (GPa)	C_{11} (Expt.)	Error (%)	C_{12} (GPa)	C_{12} (Expt.)	Error (%)	C_{44} (GPa)	C_{44} (Expt.)	Error (%)
100	120.22	131.1	8.30	89.26	98.6	9.47	46.70	49.70	6.04
200	120.32	128	6.00	88.67	97.00	8.59	46.65	47.80	2.41
300	119.15	120.32	0.97	88.67	90.64	2.17	46.26	45.81	0.98
400	117.19	118.69	1.26	86.00	90.57	5.05	46.10	43.96	4.87
500	116.68	116.94	0.22	85.71	90.38	5.17	45.94	42.11	9.10
600	114.90	115.06	0.14	85.7	90.05	4.83	45.25	40.27	12.37
700	112.83	113.02	0.17	85.11	89.59	5.00	44.59	38.43	16.03
800	110.75	110.82	0.06	83.11	88.97	6.59	43.29	36.59	18.31
900	108.32	108.45	0.12	83.00	88.18	5.87	42.59	34.76	22.53
1000	105.20	105.92	0.68	82.5	87.24	5.43	41.58	32.93	26.27

Table S17 Predicted silver elastic stiffness constants by the interatomic potential *Ag.set*

**Ag.set**									
T (K)	C_{11} (GPa)	C_{11} (Expt.)	Error (%)	C_{12} (GPa)	C_{12} (Expt.)	Error (%)	C_{44} (GPa)	C_{44} (Expt.)	Error (%)
100	112.81	131.1	13.95	86.23	98.6	12.55	43.21	49.7	13.06
200	108.05	128	15.59	83.48	97	13.94	40.65	47.8	14.96
300	104.05	120.32	13.52	80.24	90.64	11.47	38.42	45.81	16.13
400	102	118.69	14.06	79.38	90.57	12.36	37.29	43.96	15.17
500	100.2	116.94	14.32	78.3	90.38	13.37	36.25	42.11	13.92
600	98.7	115.06	14.22	77.73	90.05	13.68	35.4	40.27	12.09
700	97.85	113.02	13.42	76.77	89.59	14.31	34.47	38.43	10.30
800	94.74	110.82	14.51	75.08	88.97	15.61	33.3	36.59	8.99
900	92.85	108.45	14.38	77	88.18	12.68	32.36	34.76	6.90
1000	91.48	105.92	13.63	73.82	87.24	15.38	31.33	32.93	4.86

Table S18 Predicted silver elastic stiffness constants by the interatomic potential *Ag.eam.fs*

**Ag.eam.fs**									
T (K)	C_{11} (GPa)	C_{11} (Expt.)	Error (%)	C_{12} (GPa)	C_{12} (Expt.)	Error (%)	C_{44} (GPa)	C_{44} (Expt.)	Error (%)
100	133.72	131.1	2.00	99.48	98.6	0.89	60.01	49.7	20.74
200	132.89	128	3.82	99.40	97	2.47	61.59	47.8	28.85
300	130.82	120.32	8.73	98.62	90.64	8.80	61.59	45.81	34.45
400	128.22	118.69	8.03	97.52	90.57	7.67	62.42	43.96	41.99
500	126.35	116.94	8.05	96.55	90.38	6.83	62.15	42.11	47.59
600	125.09	115.06	8.72	95.90	90.05	6.50	62.50	40.27	55.20
700	122.21	113.02	8.13	95.16	89.59	6.22	61.67	38.43	60.47
800	118.94	110.82	7.33	93.25	88.97	4.81	61.32	36.59	67.59
900	117.00	108.45	7.88	91.67	88.18	3.96	60.12	34.76	72.96
1000	115.63	105.92	9.17	92.40	87.24	5.91	59.06	32.93	79.35

Table S19 Predicted silver elastic stiffness constants by the interatomic potential *PdAgH_MorsePd3Ag.eam.alloy*

**PdAgH_MorsePd3Ag.eam.alloy**									
T (K)	C_{11} (GPa)	C_{11} (Expt.)	Error (%)	C_{12} (GPa)	C_{12} (Expt.)	Error (%)	C_{44} (GPa)	C_{44} (Expt.)	Error (%)
100	120.14	131.1	8.36	89.48	98.6	9.25	47.30	49.7	4.83
200	120.45	128	5.90	88.64	97	8.62	47.56	47.8	0.50
300	118.91	120.32	1.17	87.10	90.64	3.91	46.80	45.81	2.16
400	116.70	118.69	1.68	85.60	90.57	5.49	46.54	43.96	5.87
500	116.38	116.94	0.48	84.41	90.38	6.61	46.08	42.11	9.43
600	114.78	115.06	0.24	84.37	90.05	6.31	45.30	40.27	12.49
700	113.45	113.02	0.38	83.34	89.59	6.98	44.56	38.43	15.95
800	110.50	110.82	0.29	81.20	88.97	8.73	43.55	36.59	19.02
900	110.45	108.45	1.84	81.41	88.18	7.68	42.71	34.76	22.87
1000	108.26	105.92	2.21	79.46	87.24	8.92	41.57	32.93	26.24

Table S20 Predicted silver elastic stiffness constants by the interatomic potential CuAg.eam.alloy

CuAg.eam.alloy									
T (K)	C_{11} (GPa)	C_{11} (Expt.)	Error (%)	C_{12} (GPa)	C_{12} (Expt.)	Error (%)	C_{44} (GPa)	C_{44} (Expt.)	Error (%)
100	120.75	131.1	7.89	90.01	98.6	8.71	46.94	49.7	5.55
200	121.14	128	5.36	89.78	97	7.44	47.17	47.8	1.32
300	118.00	120.32	1.93	86.47	90.64	4.60	46.55	45.81	1.62
400	117.54	118.69	0.97	86.19	90.57	4.84	46.18	43.96	5.05
500	115.27	116.94	1.43	85.71	90.38	5.17	45.69	42.11	8.50
600	115.08	115.06	0.02	84.47	90.05	6.20	45.26	40.27	12.39
700	114.88	113.02	1.65	84.12	89.59	6.11	44.79	38.43	16.55
800	112.08	110.82	1.14	81.65	88.97	8.23	43.47	36.59	18.80
900	108.13	108.45	0.30	80.07	88.18	9.20	42.51	34.76	22.30
1000	104.59	105.92	1.26	79.16	87.24	9.26	41.07	32.93	24.72

Figures S1 to S3:

These figures show contours of per-atom stress, common neighbor analysis and centro-symmetry, during uniaxial tensile and shear strains. It is worth to mention that color coding, ranges and values associated to each color, are different and independent for each of the uniaxial tensile and shear strains. Therefore, one may see different colors for lattice at the initial condition and after the deformations, for both types of applied strains. We did not aim to show exact color coding and ranges since it is far from the scope of this work. So, these figures only display roughly approximations for knowing the changes in the mentioned calculated contours, during the strains. As an instance, the force field *Au.eam.lammps* is applied for creating figures S1 to S3 at 300K.

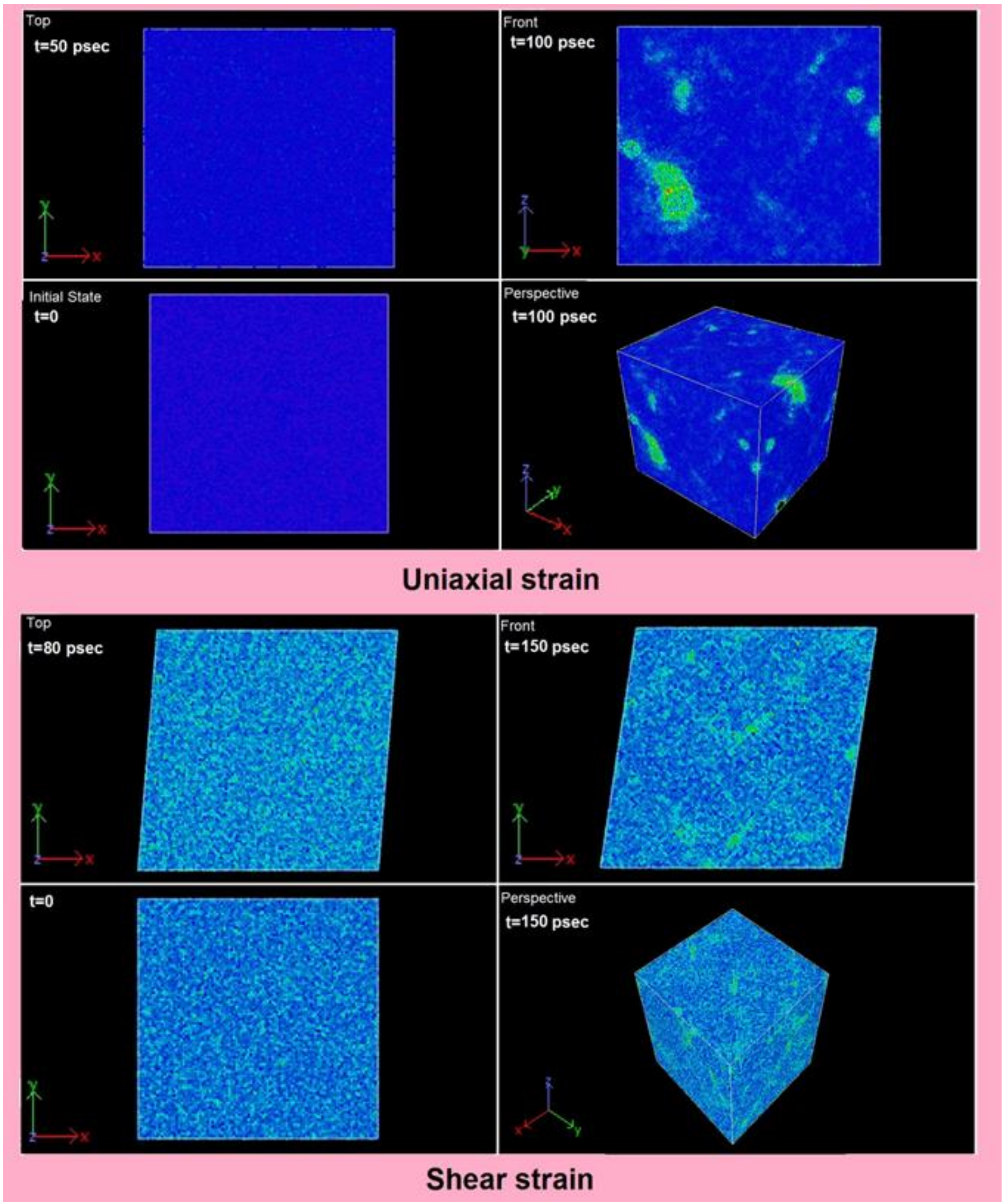

Figure S1 Per-atom stress color contour for gold single crystal during uniaxial tensile and shear strains

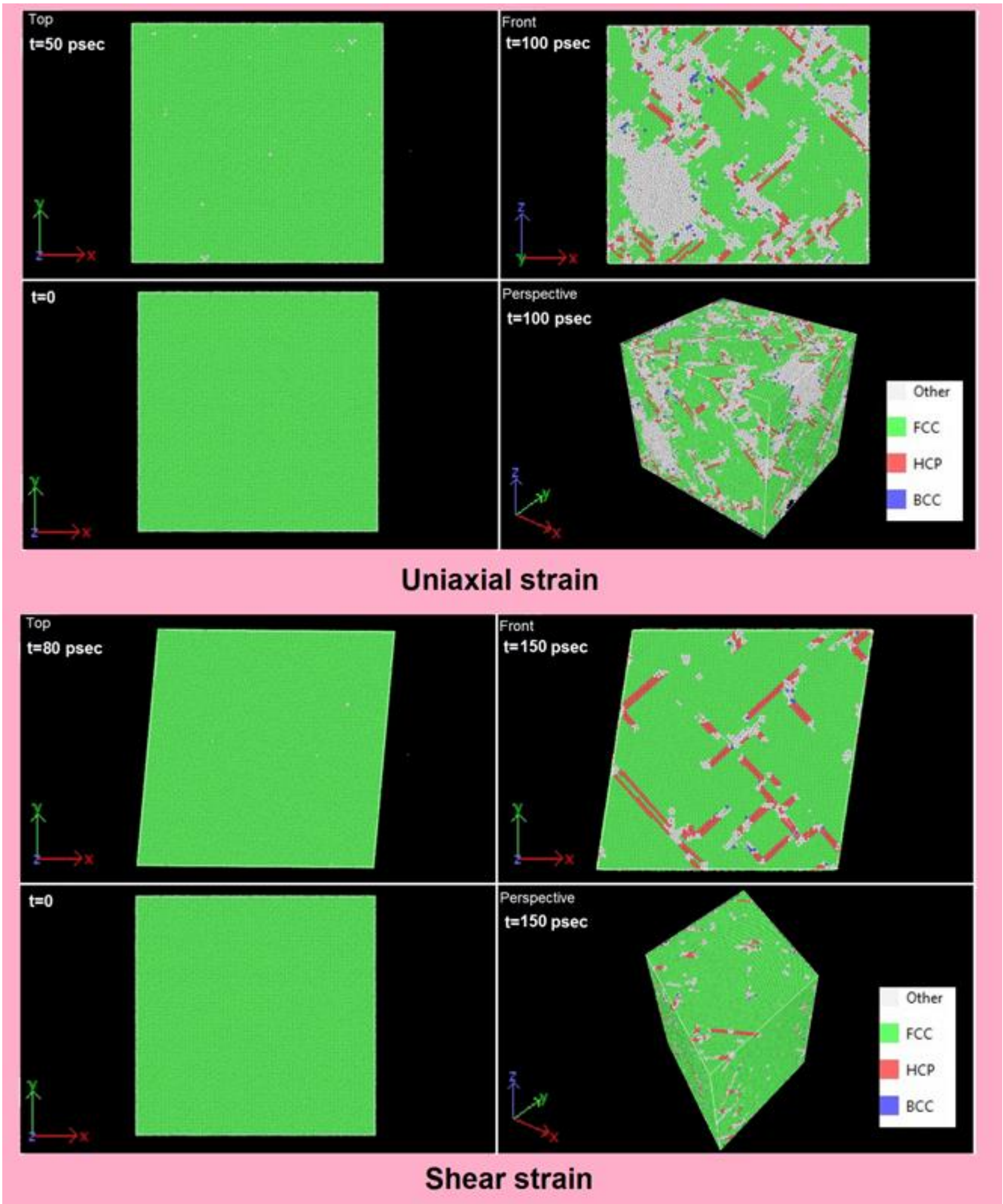

Figure S2 Common neighbor analysis for gold single crystal during uniaxial tensile and shear strains

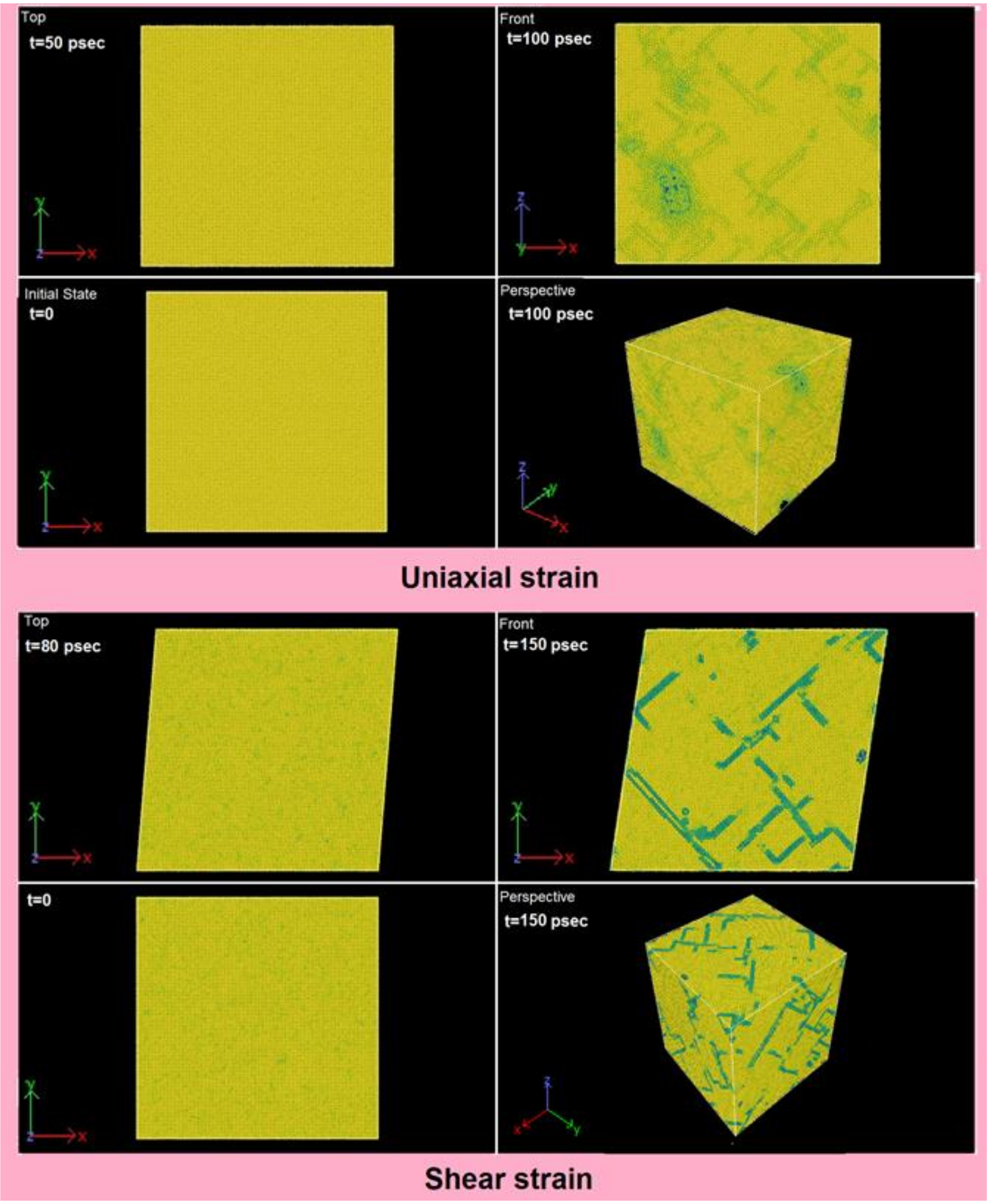

Figure S3 Centro-symmetry color contour for gold single crystal during uniaxial tensile and shear strains

Figures S4 to S12:

These figures show the discrepancies between experimental values and those arises from simulations using each interatomic potential, for predicting bulk, shear and Young's moduli of Pt, Au and Ag, using VRH model.

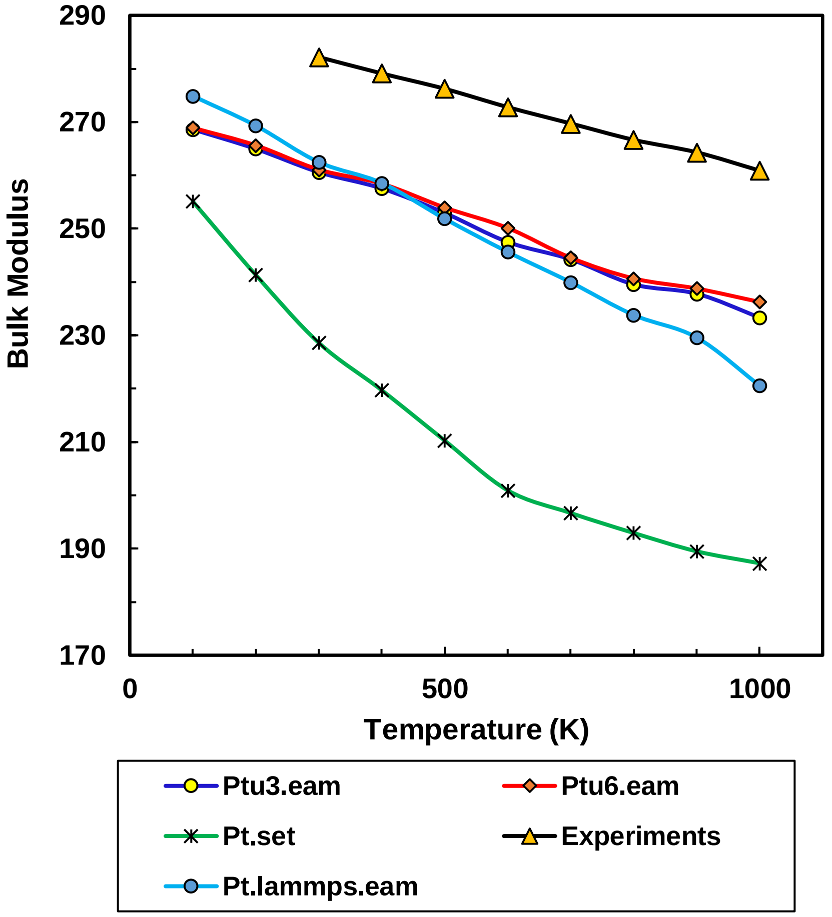

Figure S4 Platinum Bulk modulus predicted by different interatomic potentials using VRH method

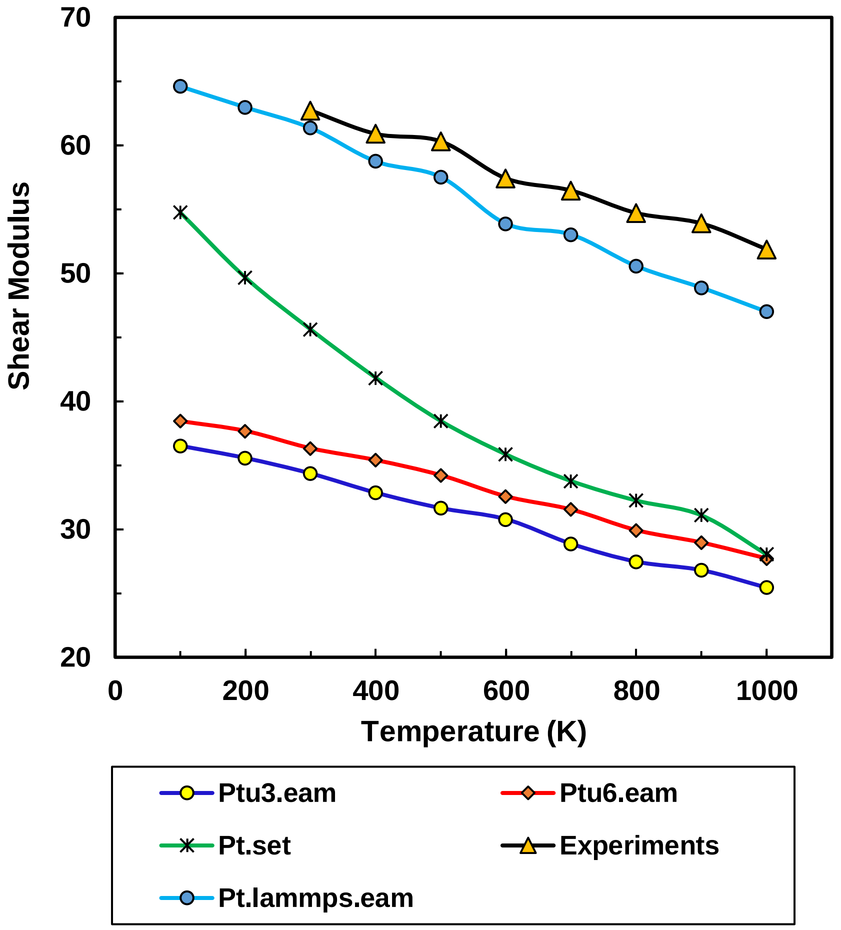

Figure S5 Platinum Shear modulus predicted by different interatomic potentials using VRH method

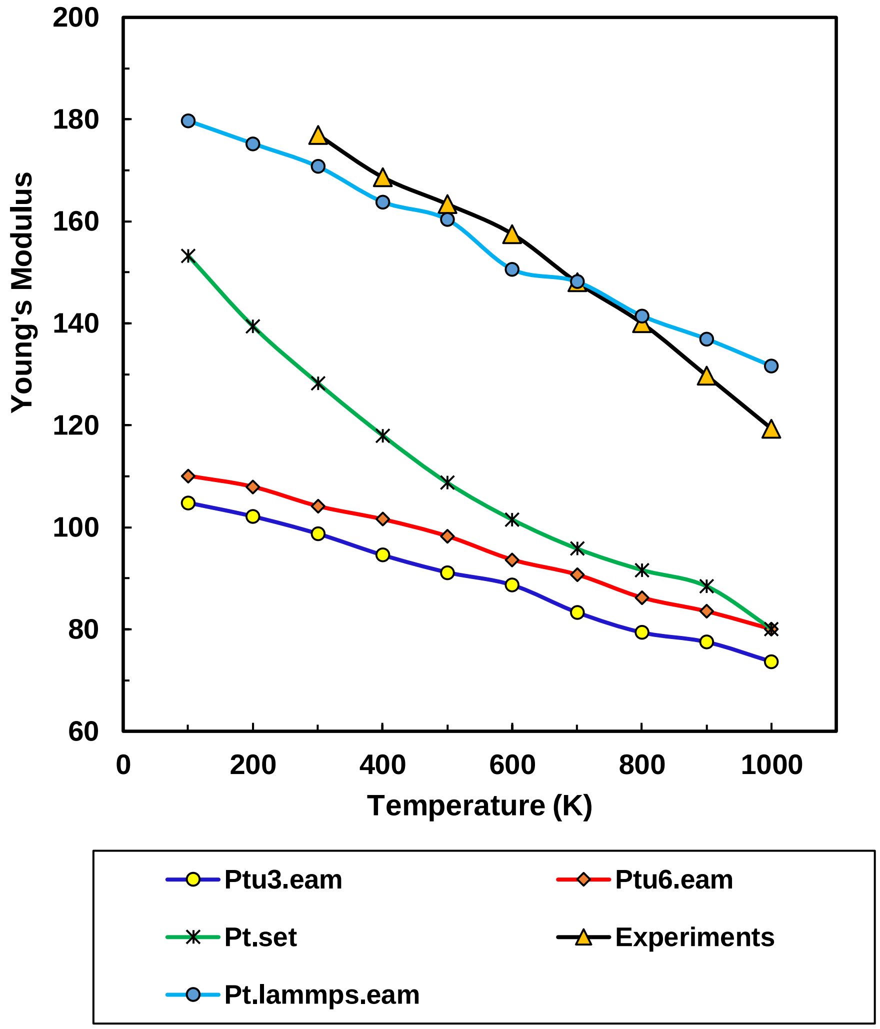

Figure S6 Platinum Young's modulus predicted by different interatomic potentials using VRH method

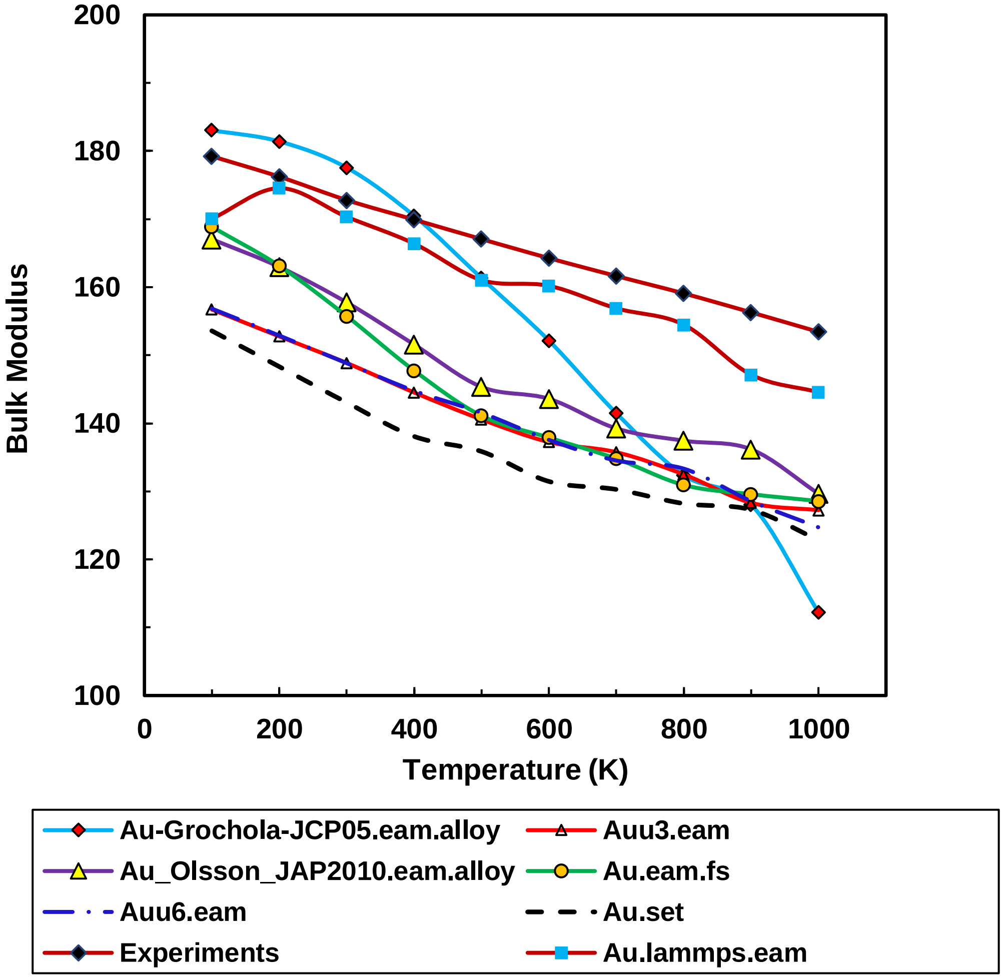

Figure S7 Gold Bulk modulus predicted by different interatomic potentials using VRH method

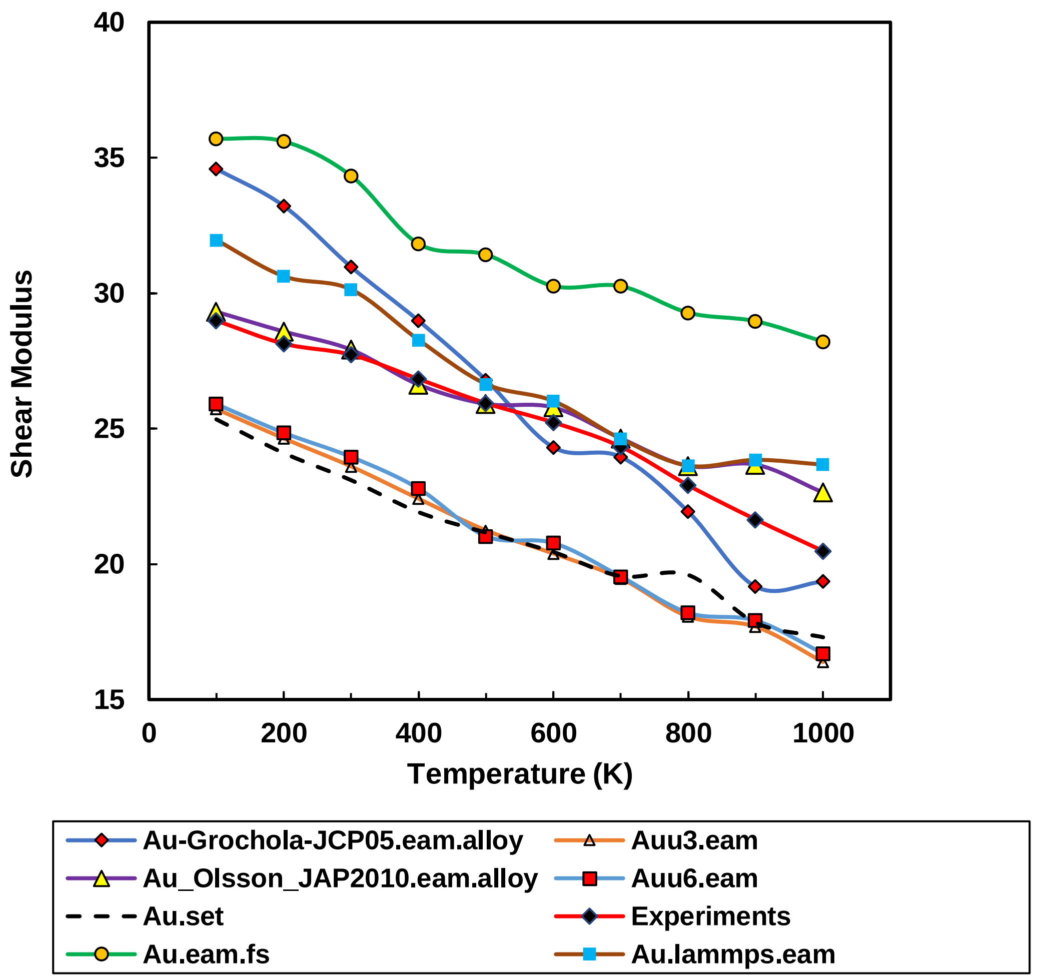

Figure S8 Gold shear modulus predicted by different interatomic potentials using VRH method

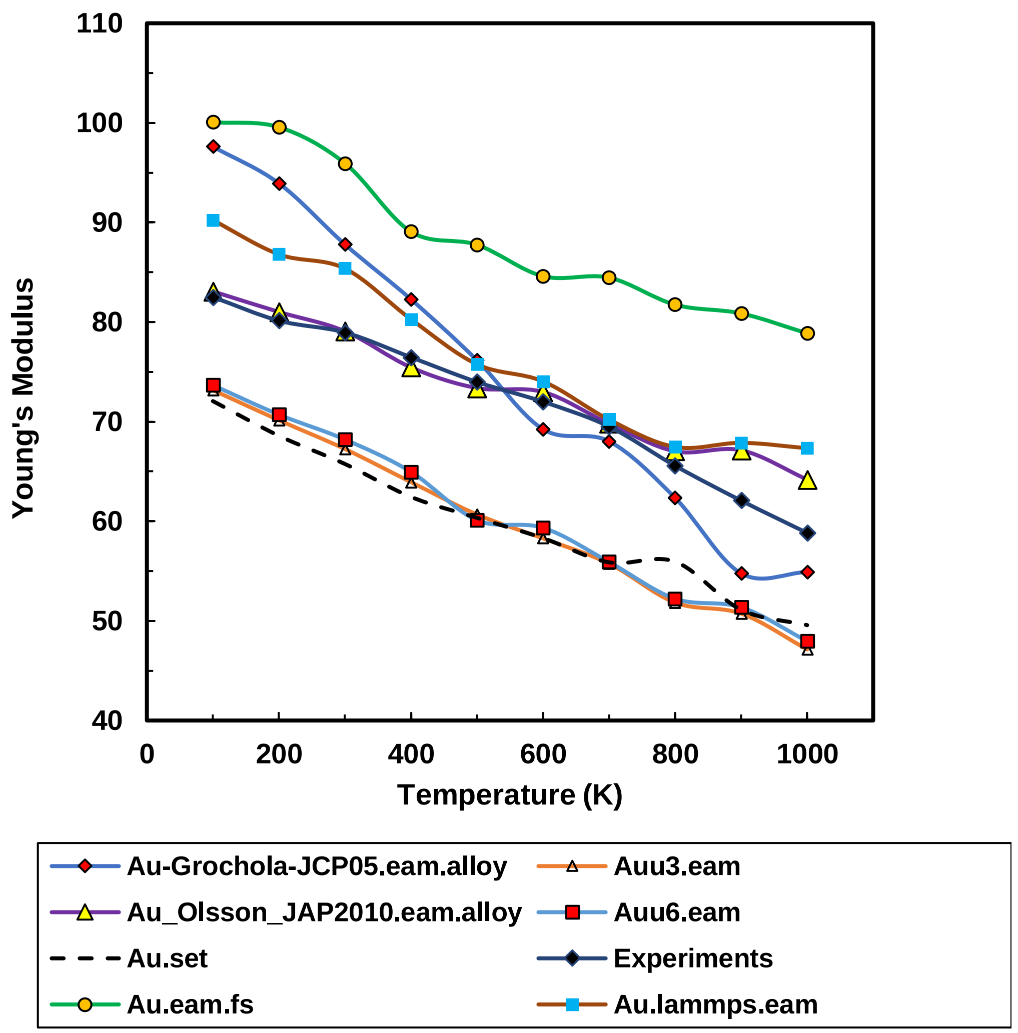

Figure S9 Gold Young's modulus predicted by different interatomic potentials using VRH method

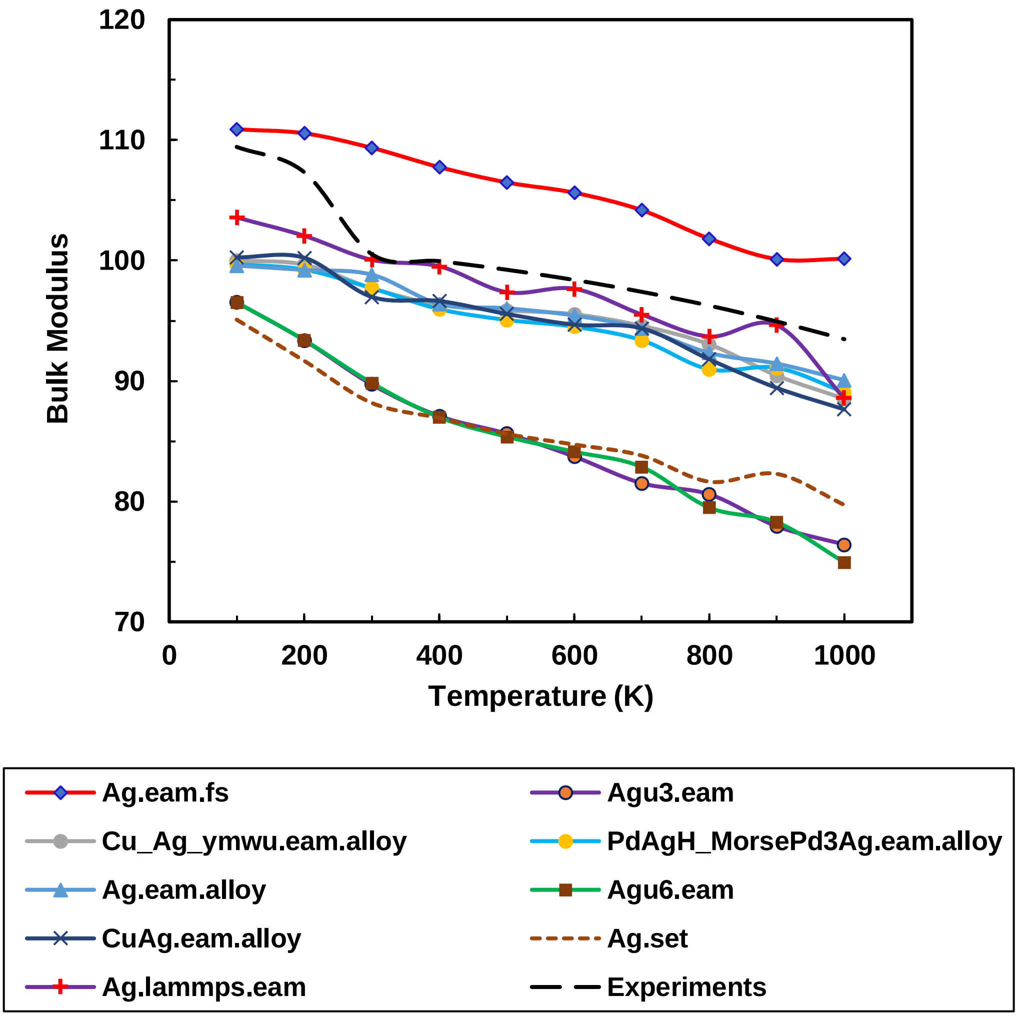

Figure S10 Silver Bulk modulus predicted by different interatomic potentials using VRH method

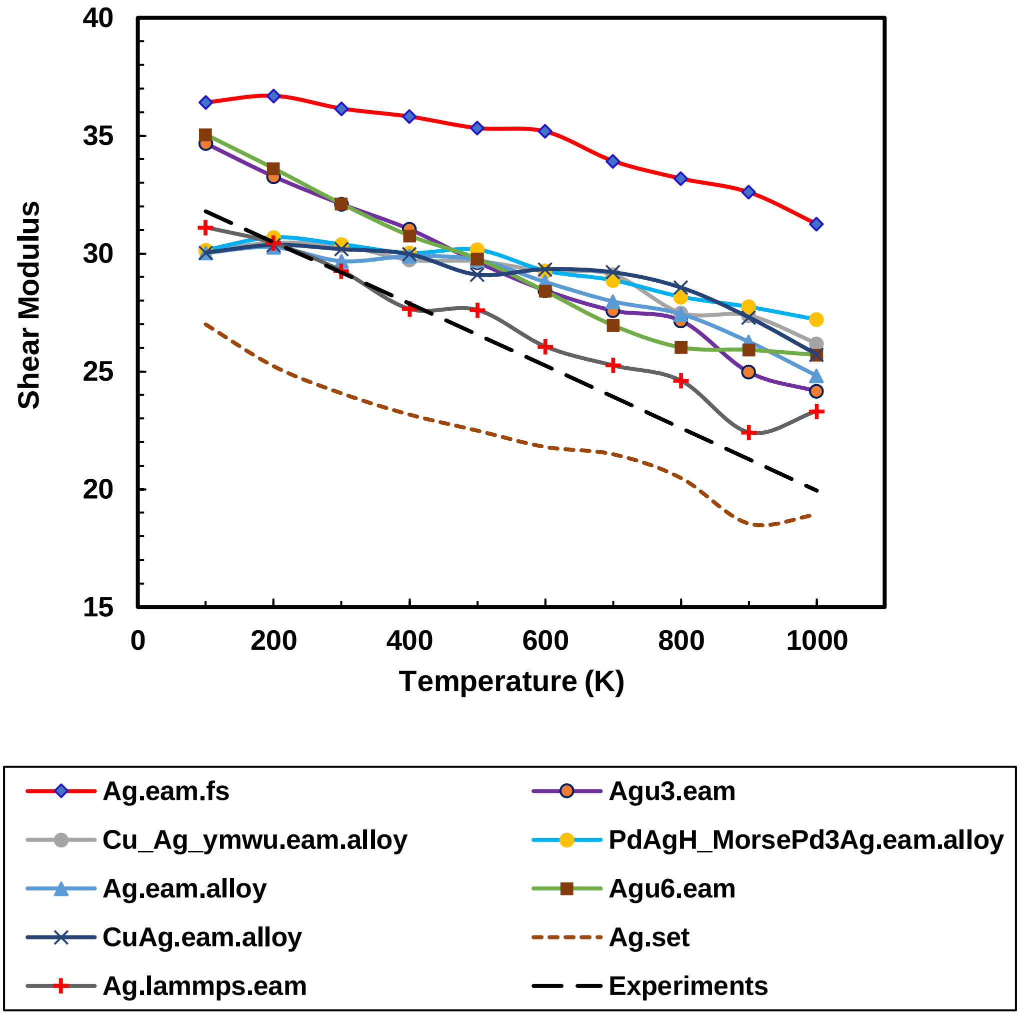

Figure S11 Silver shear modulus predicted by different interatomic potentials using VRH method

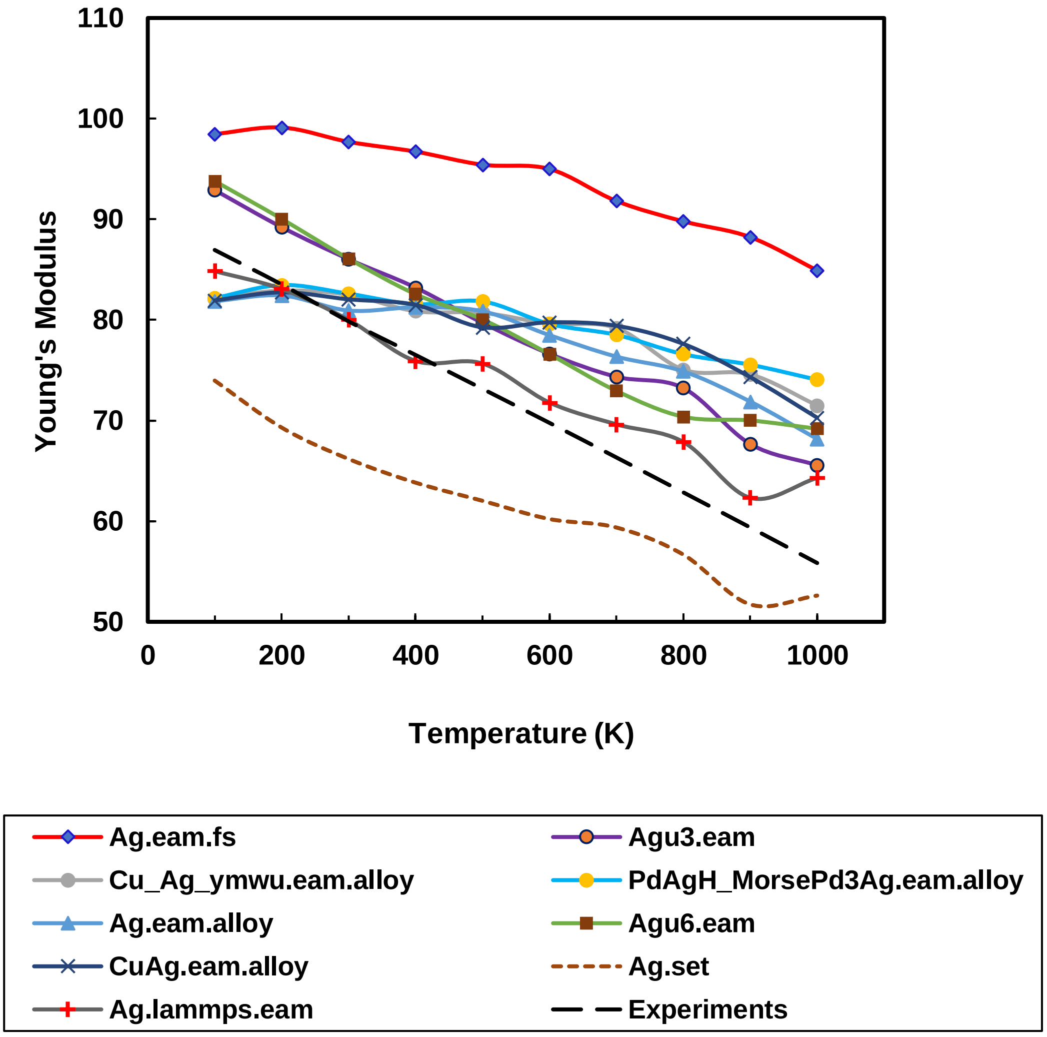

Figure S12 Silver Young's modulus predicted by different interatomic potentials using VRH method
